# Determination of effective synaptic conductances using somatic voltage clamp

**DOI:** 10.1371/journal.pcbi.1006871

**Published:** 2019-03-05

**Authors:** Songting Li, Nan Liu, Li Yao, Xiaohui Zhang, Douglas Zhou, David Cai

**Affiliations:** 1 School of Mathematical Sciences, MOE-LSC, and Institute of Natural Sciences, Shanghai Jiao Tong University, Shanghai, China; 2 State Key Laboratory of Cognitive Neuroscience and Learning, IDG/McGovern Institute for Brain Research, Beijing Normal University, Beijing, China; 3 Courant Institute of Mathematical Sciences and Center for Neural Science, New York University, New York, New York, United States of America; 4 NYUAD Institute, New York University Abu Dhabi, Abu Dhabi, United Arab Emirates; University of Edinburgh, UK, UNITED KINGDOM

## Abstract

The interplay between excitatory and inhibitory neurons imparts rich functions of the brain. To understand the synaptic mechanisms underlying neuronal computations, a fundamental approach is to study the dynamics of excitatory and inhibitory synaptic inputs of each neuron. The traditional method of determining input conductance, which has been applied for decades, employs the synaptic current-voltage (I-V) relation obtained via voltage clamp. Due to the space clamp effect, the measured conductance is different from the local conductance on the dendrites. Therefore, the interpretation of the measured conductance remains to be clarified. Using theoretical analysis, electrophysiological experiments, and realistic neuron simulations, here we demonstrate that there does not exist a transform between the local conductance and the conductance measured by the traditional method, due to the neglect of a nonlinear interaction between the clamp current and the synaptic current in the traditional method. Consequently, the conductance determined by the traditional method may not correlate with the local conductance on the dendrites, and its value could be unphysically negative as observed in experiment. To circumvent the challenge of the space clamp effect and elucidate synaptic impact on neuronal information processing, we propose the concept of effective conductance which is proportional to the local conductance on the dendrite and reflects directly the functional influence of synaptic inputs on somatic membrane potential dynamics, and we further develop a framework to determine the effective conductance accurately. Our work suggests re-examination of previous studies involving conductance measurement and provides a reliable approach to assess synaptic influence on neuronal computation.

## Author summary

To understand synaptic mechanisms underlying neuronal computations, a fundamental approach is to use voltage clamp to measure the dynamics of excitatory and inhibitory input conductances. Due to the space clamp effect, the measured conductance in general deviates from the local input conductance on the dendrites, hence its biological interpretation is questionable, as we demonstrate in this work. We further propose the concept of effective conductance that is proportional to the local input conductance on the dendrites and reflects directly the synaptic impact on spike generation, and develop a framework to determine the effective conductance reliably. Our work provides a biologically plausible metric for elucidating synaptic influence on neuronal computation under the constraint of the space clamp effect.

## Introduction

Neurons receive myriad excitatory (E) and inhibitory (I) synaptic inputs at dendrites. The spatiotemporal interaction between these E and I inputs are crucial for neuronal computation [1–3], for instance, to shape neural activity [4, 5], to enhance feature selectivity [6, 7], to modulate neural oscillations [8], and to balance network dynamics [9, 10]. To understand synaptic mechanisms underlying neuronal computation, it is important to investigate the dynamics of the pure E and I inputs to a neuron via electrophysiological recording techniques. Somatic voltage clamp has become a popular approach to achieve this both *in vitro* and *in vivo* studies over the last thirty years [11]. For instance, voltage clamp has been extensively applied to areas including visual [12–14], auditory [4, 15–17], and prefrontal cortex [18, 19].

To reveal the quantitative information of E and I inputs, data collected in voltage clamp mode needs to be further processed to determine the input conductance values. In the traditional method, the dynamics of the neuronal voltage is described as [20]

$$\begin{array}{c} c\frac{dV}{dt}=-g_{L}\left(V-\varepsilon_{L}\right)-g_{E}\left(V-\varepsilon_{E}\right)-g_{I}\left(V-\varepsilon_{I}\right)+I_{inj}, \end{array}$$ (1)

where *c* is the membrane capacitance, *V* is the membrane potential, *g*_*L*_, *g*_*E*_ and *g*_*I*_ are the leak, E, and I conductances, respectively, *ε*_*L*_, *ε*_*E*_ and *ε*_*I*_ are the corresponding reversal potentials, respectively, and *I*_*inj*_ is the externally injected current. Here all potentials are relative to the resting potential. Using the voltage clamp to hold the somatic voltage *V* at different levels, i.e., $c\frac{dV}{dt}=0$, one can obtain the corresponding synaptic currents $I_{syn}^{inj}=g_{E}\left(\varepsilon_{E}-V\right)+g_{I}\left(\varepsilon_{I}-V\right)$ (the superscript “inj” emphasizes that the synaptic current is measured in the presence of injected current given by the voltage clamp) and linearly fit an I-V relation at each time point. By casting $I_{syn}^{inj}$ as $I_{syn}^{inj}=-kV+b$, the slope

$$\begin{array}{c} k=g_{E}+g_{I} \end{array}$$ (2)

is the total conductance (the linear summation of the E and I conductances) and the intercept

$$\begin{array}{c} b=g_{E}\varepsilon_{E}+g_{I}\varepsilon_{I} \end{array}$$ (3)

is the reversal current (the weighted summation of the E and I conductances). Therefore, by measuring the slope and the intercept of the I-V relation, one can solve Eqs 2 and 3 to obtain the values of *g*_*E*_ and *g*_*I*_.

Despite the extensive application of voltage clamp to determine E and I conductances for decades, it has yet to address various important issues related to the validity of the above approach. First, in Eq 1, is the assumption of the linear summation of the synaptic current from the dendrites and the injected clamp current from the soma valid in a neuron with spatial dendrites? Second, in the presence of the space clamp effect [21–27], the membrane potential at distal synapses can deviate greatly from the holding potential and the E and I conductances obtained from Eqs 2 and 3 in the traditional method can be distorted significantly from the synaptic conductances on the dendrites. How to interpret the value of the conductance measured using the traditional method? Whether there is a direct relation between the measured conductance and the local conductance that allows one to assess synaptic influence on neuronal computation? Third, if the measured conductance does not correlate with the local conductance, then how to characterize synaptic impact on neuronal computation under the constraint of the space clamp effect?

Using theoretical analysis, electrophysiological experiments, and realistic neuron simulations, here we demonstrate that there does not exist a transform between the local conductance and the conductance measured by the traditional method because of the neglect of a *nonlinear interaction* between clamp current at the soma and synaptic currents from the dendrites in the traditional method. Consequently, the conductance determined by the traditional method may not correlate with the local conductance, and it could give unphysically negative value as observed in experiments. Under the constraint of the space clamp effect, we propose the concept of *effective conductance*, which reflects directly the functional impact of synaptic inputs on action potential initiation and thereby neuronal information processing. We then devise a framework for determining the effective conductance and accordingly verify it in both electrophysiological experiments and realistic neuron simulations, thereby establishing a biologically plausible metric for elucidating synaptic impact on neuronal computation. We discuss the scientific advance of our study in contrast to existing studies addressing the space-clamp effect and issues relevant to the application of our method in the section of Discussion.

## Materials and methods

### Slice electrophysiology

The preparation of acute hippocampal slices (350 *μ*m thick) from Sprague Dawley rats of postnatal days 15-20 followed a method described in our previous study [28]. The animal experimental protocol was approved by the Animal Use and Care Committee of State Key Laboratory of Cognitive Neuroscience & Learning at Beijing Normal University (IACUC-BNU-NKLCNL-2016-02). In brief, rats were deeply anesthetized by i.p. injection of pentobarbital (30 mg/kg weight), and the brain was quickly dissected and then incubated in the ice-cold artificial cerebrospinal fluid (aCSF), which was oxygenated with 95% O_2_ / 5% CO_2_. Coronal hippocampal slices were sectioned with vibratome (VT1200, Leica) and incubated in oxygenated aCSF at 34 °C for 30 min, followed by an incubation at 20-22 °C till the use for the electrophysiological recording. The aCSF contained (in mM) 125 NaCl, 3 KCl, 2 CaCl_2_, 2 MgSO_4_, 1.25 NaH_2_PO_4_, 1.3 sodium ascorbate, 0.6 sodium pyruvate, 26 NaHCO_3_, and 11 D-glucose (pH 7.4 bubbled with 95% O_2_ / 5% CO_2_).

Whole-cell recording was made on the hippocampal CA1 pyramidal cell (PC) in slices in a chamber perfused with the same aCSF solution as that used for the brain slicing (2 ml/min; 30-32°C), under an Olympus upright microscope (BX51WI) that was equipped with the differential interference contrast (DIC) and fluorescence optics as well as an infrared camera (IR-1000E, DAGE-MTI). The borosilicate-glass micropipettes were pulled by a Sutter puller (P-1000) and filled by an internal solution containing (in mM) 145 K-gluconate, 5 KCl, 10 HEPES, 10 disodium phosphocreatine, 4 Mg-ATP, 0.3 Na-GTP and 0.2 EGTA (pH 7.3, 295 mOsm). Simultaneous recordings from the cell body and dendrite of a PC followed a procedure reported previously [29], in which whole cell recording on the soma was first made using a micropipette (3-5 MΩ; with 20 *μ*M Alexa Fluor 488, InvitroGene), followed by another recording on Alexa Flour 488 (green)-labeled apical dendritic arbor at position ∼100 *μ*m away from the soma with a micropipette (10-15 MΩ, filled with the internal solution without Alexa Fluor 488). The serial resistance was compensated by >90% using the built-in function of the amplifier MultiClamp 700B (Molecular Devices). Holding potentials of recorded cells were corrected for a calculated liquid junction potential [30] of ∼15 mV. In the dynamic clamp recording experiments, either AMPA type glutamate receptor-mediated excitatory conductance or GABA_A_ receptor-mediated inhibitory conductance was intracellularly injected to the recorded PCs through the whole-cell recording pipette, using the built-in dynamic-clamp function of a 1401 Power3 digitizer (CED) and the Spike2 software (v5.08; CED). Kinetics of AMPA or GABA_A_ receptor conductance were in the form of two exponential functions with different rise/decay time constants: 5/7.8 ms for AMPA conductance; 6/18 ms for GABA_A_ conductance. Their respective reversal potentials, *E*_*AMPA*_ and $E_{GABA_{A}}$, were set as 0 mV and −70 mV. Membrane voltage or current signals were amplified with a MultiClamp 700B amplifier (Molecular Devices), filtered at10 KHz (low-pass), digitalized by an analog-digital converter (1401 Power3, CED) at 50 KHz, and then acquired by the Spike2 software into a computer for further analysis.

The experimental data of a sample neuron is available at https://github.com/songting858/Intercept-Method-code.

The laboratory protocols used in this study has been uploaded to protocols.io, http://dx.doi.org/10.17504/protocols.io.wm4fc8w.

### Realistic neuron simulation

We adapted the multi-compartment neuron model used in our previous studies [28, 31, 32] for our realistic pyramidal neuron simulation. The morphology of the reconstructed pyramidal neuron, which includes 200 compartments, was obtained from the Duke-Southampton Archive of Neuronal Morphology [33]. The passive cable properties and the densities of active conductances in the neuron model were based on published experimental data obtained from the hippocampal and cortical pyramidal neurons [34–46], and the passive cable properties were slightly tuned to capture the distance-dependent space clamp effect measured in an experiment [26] (S1 Fig). In particular, the multi-compartment neuron model included the voltage-gated sodium channel, the delayed rectifier potassium channel, two variants of A-type potassium channel, and the hyperpolarization activated channel. The synaptic inputs were given through AMPA receptor with rise/decay time constants: 5/7.8 ms and GABA_A_ receptor with rise/decay time constants: 6/18 ms. The resting potential was set to *V*_*r*_ = −70 mV, and the E and I reversal potentials were set to *E*_*AMPA*_ = 0 mV, $E_{GABA_{A}}=-80\;\text{mV}$. We used the NEURON software Version 7.4 [47] to simulate the model with time step of 0.1 ms. The detailed model description and the simulation code are available at https://github.com/songting858/Intercept-Method-code.

### Static transfer resistance analysis

We generalize the static two-port analysis [20, 28] to study the property of effective conductance, to determine theoretically the effective conductance, and to illustrate the deficiency of the traditional method in the determination of conductance due to the neglect of a nonlinear interaction between clamp current at the soma and synaptic currents from the dendrites. Note that the purpose of this analysis is to provide insights into the issue of conductance measurement. Therefore, for the sake of simplicity, we focus on the case of time-independent synaptic inputs in the analysis. The case of time-dependent synaptic inputs will be demonstrated in both electrophysiological experiments of rat CA1 pyramidal neurons and simulations of the realistic pyramidal neuron model in the section of Results.

#### Definition of effective conductance

We first introduce the concept of effective conductance. Experiments have shown that, although a neuron is not an electrically compact point, the dynamics of its somatic membrane potential in response to current injection can be well characterized by a point leaky integrator [48, 49]. Based on this fact, it is natural to consider the soma as a point and accordingly, we introduce the concept of effective conductance at the soma, which is defined, by Ohm’s law, as the ratio of the synaptic current $I_{q}^{eff}$ arriving at the soma to the driving force (difference between the reversal potential *ε*_*q*_ and the somatic membrane potential *V*_*S*_) in the presence of either E or I input, i.e.,

$$\begin{array}{c} g_{q}^{eff}=\frac{I_{q}^{eff}}{\varepsilon_{q}-V_{S}}, \end{array}$$ (4)

where *q* = *E*, *I*. It should be stressed that, in order to distinguish from the synaptic current measured using voltage clamp in the traditional method, $I_{q}^{eff}$ is the synaptic current arriving at the soma in the absence of any externally injected current.

#### Derivation of effective conductance

We derive the relationship between the effective conductance measured at the soma and the local synaptic conductance induced at synapses on the dendrite. If a neuron receives an E input on the dendrite, the local synaptic current on the dendrite can be characterized by Ohm’s law,

$$\begin{array}{c} I_{E}=g_{E}\left(\varepsilon_{E}-V_{E}\right), \end{array}$$ (5)

where *g*_*E*_ is the local E conductance at the synapse, *ε*_*E*_ is the E reversal potential, and *V*_*E*_ is the local membrane potential at the synapse. Unless otherwise specified, all potentials are relative to the resting potential.

Based on Ohm’s law, the local membrane potential *V*_*E*_ can be computed by

$$\begin{array}{c} V_{E}=K_{EE}I_{E}, \end{array}$$ (6)

where *K*_*EE*_ is the resistance at the E synapse. Therefore, combining Eqs 5 and 6, the local membrane potential *V*_*E*_ is expressed as

$$\begin{array}{c} V_{E}=\frac{g_{E}K_{EE}\varepsilon_{E}}{1+g_{E}K_{EE}}. \end{array}$$ (7)

Similarly, the membrane potential measured at the soma *V*_*S*_ in response to the synaptic input *g*_*E*_ can be computed by

$$\begin{array}{c} V_{S}=K_{ES}I_{E}, \end{array}$$ (8)

where *K*_*ES*_ is the transfer resistance between the E synapse and the soma. The combination of Eqs 5–8 yields the somatic membrane potential in response to *g*_*E*_ on the dendrite,

$$\begin{array}{c} V_{S}=\frac{g_{E}K_{ES}\varepsilon_{E}}{1+g_{E}K_{EE}}. \end{array}$$ (9)

Now if we denote the E synaptic current arriving at the soma as $I_{E}^{eff}$, which is termed as the effective E synaptic current and is induced by the synaptic current *I*_*E*_ (Eq 5) from the dendrite, then by definition, the effective E conductance denoted by $g_{E}^{eff}$ satisfies

$$\begin{array}{c} I_{E}^{eff}=g_{E}^{eff}\left(\varepsilon_{E}-V_{S}\right), \end{array}$$ (10)

where the superscript “*eff*” emphasizes the effective quantities at the soma.

Ohm’s law links the somatic membrane potential with the somatic current as

$$\begin{array}{c} V_{S}=K_{SS}I_{E}^{eff}, \end{array}$$ (11)

where *K*_*SS*_ is the resistance at the soma.

From Eqs 10 and 11, we can obtain an expression for the effective E conductance $g_{E}^{eff}$ at the soma,

$$\begin{array}{c} g_{E}^{eff}=\frac{V_{S}}{K_{SS}\left(\varepsilon_{E}-V_{S}\right)}. \end{array}$$ (12)

Because *ε*_*E*_ is relatively large compared with *V*_*S*_, using Taylor expansion, Eq 12 can be approximated as

$$\begin{array}{c} g_{E}^{eff}=\frac{V_{S}}{K_{SS}\varepsilon_{E}}(1+\frac{V_{S}}{\varepsilon_{E}}+o(\frac{V_{S}}{\varepsilon_{E}})). \end{array}$$ (13)

Substituting Eq 9 into Eq 13, and assuming that *g*_*E*_ is small, we have the effective E conductance at the soma,

$$\begin{array}{c} g_{E}^{eff}=\frac{K_{ES}}{K_{SS}}g_{E}+o\left(g_{E}\right), \end{array}$$ (14)

where *o*(*g*_*E*_) includes all high-order terms of the local conductance *g*_*E*_.

Similarly, we can derive an expression for the effective I conductance at the soma,

$$\begin{array}{c} g_{I}^{eff}=\frac{K_{IS}}{K_{SS}}g_{I}+o\left(g_{I}\right), \end{array}$$ (15)

where *o*(*g*_*I*_) includes all high-order terms of the local conductance *g*_*I*_. In the derivation of Eq 15, we assume that, when the neuron receives an I input on its dendrite, the ratio from the somatic voltage response $V_{S}^{I}$ and the inhibitory reversal potential *ε*_*I*_ denoted as $\frac{V_{S}^{I}}{\varepsilon_{I}}$ shall be relatively small. Although the validity of this condition is not as obvious as that in the case of an E input, it could often happen *in vivo* and *in vitro*. In particular, when a neuron receives balanced E and I inputs as observed in many experiments, i.e., *g*_*I*_ is the same order as *g*_*E*_, $\frac{V_{S}^{I}}{\varepsilon_{I}}$ can be proven to be equally small as $\frac{V_{S}^{E}}{\varepsilon_{E}}$ as follows. Based on Eq 9, we have $\frac{V_{S}^{E}}{\varepsilon_{E}}=\frac{g_{E}K_{ES}}{1+g_{E}K_{EE}}$ and similarly $\frac{V_{S}^{I}}{\varepsilon_{I}}=\frac{g_{I}K_{IS}}{1+g_{I}K_{II}}$, where $V_{S}^{E}$ and $V_{S}^{I}$ are the somatic voltage change in response to an E input and an I input received alone, respectively. Because the transfer functions *K*_*XY*_ for *X*, *Y* = {*E*, *I*, *S*} are location dependent but not input-type dependent, we have *K*_*ES*_ ≈ *K*_*IS*_ and *K*_*EE*_ ≈ *K*_*II*_ when the locations of the E and I inputs are close. Therefore, when *g*_*I*_ is the same order as *g*_*E*_, $\frac{V_{S}^{I}}{\varepsilon_{I}}$ shall also be the same order as $\frac{V_{S}^{E}}{\varepsilon_{E}}$. The validity of Eq 15 has also been assessed numerically even when $V_{S}^{I}$ is about the same order as *ε*_*I*_. When setting the reversal potential *ε*_*I*_ to be −10 mV, the error of the first order approximation in Eq 15 is about 9% when an IPSP is as large as −3 mV.

Therefore, to the first order accuracy of *g*_*E*_ and *g*_*I*_, the effective conductance is a proportional indicator of the local synaptic conductance induced on the dendrite.

#### Determination of effective conductance using voltage clamp

We now demonstrate theoretically how to determine the effective conductances using voltage clamp when a neuron receives both E and I inputs on the dendrite.

By Ohm’s law, we have the local E and I synaptic currents on the dendrite

$$\begin{array}{c} I_{E}=g_{E}\left(\varepsilon_{E}-V_{E}\right), \end{array}$$ (16)

$$\begin{array}{c} I_{I}=g_{I}\left(\varepsilon_{I}-V_{I}\right). \end{array}$$ (17)

Meanwhile, we can describe the local membrane potentials measured at the E synapse, the I synapse, and the soma as follows,

$$\begin{array}{c} V_{E}=K_{EE}I_{E}+K_{IE}I_{I}+K_{SE}I_{inj}, \end{array}$$ (18)

$$\begin{array}{c} V_{I}=K_{EI}I_{E}+K_{II}I_{I}+K_{SI}I_{inj}, \end{array}$$ (19)

$$\begin{array}{c} V_{S}=K_{ES}I_{E}+K_{IS}I_{I}+K_{SS}I_{inj}. \end{array}$$ (20)

Here somatic voltage clamp is applied to hold the somatic membrane potential *V*_*S*_ at a fixed level by injecting current *I*_*inj*_ at the soma. The transfer resistance between locations A and B (*K*_*AB*_) is the ratio of the voltage change in location B to the magnitude of the injected current in location A. These transfer resistances possess a reciprocal relation, i.e., the symmetric property, *K*_*XY*_ = *K*_*YX*_, which is used in our calculation. It can be rigorously proved that the symmetric property is valid for any linear system, e.g., a neuron is composed of passive cables [20]. For a neuron with active ion channels, we resort to numerical simulations to verify this symmetry property. As shown in S2 Fig, the symmetric property also holds approximately in an active neuron with nonlinear ion channel dynamics. Next, to obtain a relation between the somatic voltage and the synaptic current in the presence of the injected current, we solve Eqs 16–20.

The somatic voltage can be obtained as

$$\begin{array}{ccc} V_{S} & = & \frac{g_{E}\varepsilon_{E}(K_{ES}+g_{I}\left(K_{ES}K_{II}-K_{EI}K_{IS}\right))+g_{I}\varepsilon_{I}(K_{IS}+g_{E}\left(K_{IS}K_{EE}-K_{EI}K_{ES}\right))}{1+g_{E}K_{EE}+g_{I}K_{II}+g_{E}g_{I}\left(K_{EE}K_{II}-K_{EI}^{2}\right)}\\ & + & \frac{I_{inj}(K_{SS}+g_{E}\left(K_{EE}K_{SS}-K_{ES}^{2}\right)+g_{I}\left(K_{II}K_{SS}-K_{IS}^{2}\right))}{1+g_{E}K_{EE}+g_{I}K_{II}+g_{E}g_{I}\left(K_{EE}K_{II}-K_{EI}^{2}\right)}\\ & - & \frac{I_{inj}g_{E}g_{I}\left(K_{ES}^{2}K_{II}+K_{EE}K_{IS}^{2}+K_{EI}^{2}K_{SS}-K_{EE}K_{II}K_{SS}-2K_{EI}K_{ES}K_{IS}\right)}{1+g_{E}K_{EE}+g_{I}K_{II}+g_{E}g_{I}\left(K_{EE}K_{II}-K_{EI}^{2}\right)}. \end{array}$$ (21)

On the other hand, under voltage clamp mode, the total current at the soma leads to the somatic voltage fixed at *V*_*S*_,

$$\begin{array}{c} K_{SS}\left(I_{syn}^{inj}+I_{inj}\right)=V_{S}. \end{array}$$

Therefore, the synaptic current $I_{syn}^{inj}$ at the soma in the presence of the injected current can be obtained as

$$\begin{array}{ccc} & I_{syn}^{inj} & =\frac{V_{S}}{K_{SS}}-I_{inj}\\ & = & -\frac{g_{E}K_{ES}^{2}+g_{I}K_{IS}^{2}-g_{E}g_{I}\left(2K_{EI}K_{ES}K_{IS}-K_{ES}^{2}K_{II}-K_{IS}^{2}K_{EE}\right)}{K_{SS}+g_{E}K_{EE}K_{SS}+g_{I}K_{II}K_{SS}+g_{E}g_{I}\left(K_{EE}K_{II}K_{SS}-K_{EI}^{2}K_{SS}\right)}I_{inj}\\ & + & \frac{g_{E}\varepsilon_{E}K_{ES}+g_{I}\varepsilon_{I}K_{IS}+g_{E}g_{I}(\varepsilon_{E}\left(K_{II}K_{ES}-K_{EI}K_{IS}\right)+\varepsilon_{I}\left(K_{EE}K_{IS}-K_{EI}K_{ES}\right))}{K_{SS}+g_{E}K_{EE}K_{SS}+g_{I}K_{II}K_{SS}+g_{E}g_{I}\left(K_{EE}K_{II}K_{SS}-K_{EI}^{2}K_{SS}\right)}, \end{array}$$ (22)

We note that the synaptic current in Eq 22 depends on the injected current, and the multiplication between the synaptic conductances and the injected current indicates a *nonlinear interaction* between the synaptic currents from the dendrite and the injected current at the soma. We also note that both *V*_*S*_ (in Eq 21) and $I_{syn}^{inj}$ (in Eq 22) are linear functions of *I*_*inj*_, therefore, we can find a linear relationship between *V*_*S*_ and $I_{syn}^{inj}$

$$\begin{array}{c} I_{syn}^{inj}=-kV_{S}+b, \end{array}$$ (23)

which holds for arbitrary *I*_*inj*_, and the corresponding holding potential *V*_*S*_.

By comparing the coefficients of *I*_*inj*_ on both sides of Eq 23, we can obtain the expression for the slope *k* and the intercept *b* as follows,

$$\begin{array}{c} k=\frac{g_{E}K_{ES}^{2}+g_{I}K_{IS}^{2}+g_{E}g_{I}\left(K_{ES}^{2}K_{II}+K_{IS}^{2}K_{EE}-2K_{EI}K_{ES}K_{IS}\right)}{K_{SS}(K_{SS}+g_{E}\left(K_{EE}K_{SS}-K_{ES}^{2}\right)+g_{I}\left(K_{II}K_{SS}-K_{IS}^{2}\right)+g_{E}g_{I}K_{EIS})},\\ b=\frac{g_{E}\varepsilon_{E}K_{ES}+g_{I}\varepsilon_{I}K_{IS}+g_{E}g_{I}(\varepsilon_{E}\left(K_{ES}K_{II}-K_{EI}K_{IS}\right)+\varepsilon_{I}\left(K_{IS}K_{EE}-K_{EI}K_{ES}\right))}{K_{SS}+g_{E}\left(K_{EE}K_{SS}-K_{ES}^{2}\right)+g_{I}\left(K_{II}K_{SS}-K_{IS}^{2}\right)+g_{E}g_{I}K_{EIS}}, \end{array}$$

where $K_{EIS}=K_{EE}K_{II}K_{SS}+2K_{EI}K_{ES}K_{IS}-K_{ES}^{2}K_{II}-K_{IS}^{2}K_{EE}-K_{EI}^{2}K_{SS}$.

To the first order accuracy of *g*_*E*_ and *g*_*I*_, the expressions of *k* and *b* reduce to

$$\begin{array}{ccc} k & = & {\left(\frac{K_{ES}}{K_{SS}}\right)}^{2}g_{E}+{\left(\frac{K_{IS}}{K_{SS}}\right)}^{2}g_{I}+o\left(g\right)\\ & = & (\frac{K_{ES}}{K_{SS}})g_{E}^{eff}+(\frac{K_{IS}}{K_{SS}})g_{I}^{eff}+o\left(g\right), \end{array}$$ (24)

$$\begin{array}{ccc} b & = & (\frac{K_{ES}}{K_{SS}})g_{E}\varepsilon_{E}+(\frac{K_{IS}}{K_{SS}})g_{I}\varepsilon_{I}+o\left(g\right)\\ & = & g_{E}^{eff}\varepsilon_{E}+g_{I}^{eff}\varepsilon_{I}+o\left(g\right). \end{array}$$ (25)

The above results show that the slope of the I-V relation does not equal the total effective conductance but the intercept equals the effective reversal current. Therefore, the I-V relation allows only the value of the intercept *b* to measure the effective E and I conductances but not the value of the slope *k* which contains generally unknown factors *K*_*ES*_/*K*_*SS*_ and *K*_*IS*_/*K*_*SS*_. Because we have two unknown variables $g_{E}^{eff}$ and $g_{I}^{eff}$ here, in order to solve them, a possible solution is to provide at least two equations of I-V relations. For example, we can vary the I reversal potential from *ε*_*I*_ to $\varepsilon_{I}^{{}^{\prime }}$ to obtain a second intercept equation

$$\begin{array}{c} b^{{}^{\prime }}=g_{E}^{eff}\varepsilon_{E}+g_{I}^{eff}\varepsilon_{I}^{{}^{\prime }}, \end{array}$$ (26)

and then the effective E and I conductances can be obtained by solving Eqs 25 and 26.

#### Error estimation for conductance determined by the traditional method

Here we estimate the error of the effective conductance determined by the traditional method. From Eqs 24 and 25, the effective E and I conductances can be expressed as

$$\begin{array}{c} g_{E}^{eff}=\frac{b\left(\frac{K_{IS}}{K_{SS}}\right)+k\varepsilon_{I}}{\left(\frac{K_{ES}}{K_{SS}}\right)\varepsilon_{I}-\left(\frac{K_{IS}}{K_{SS}}\right)\varepsilon_{E}},\\ g_{I}^{eff}=\frac{b\left(\frac{K_{ES}}{K_{SS}}\right)+k\varepsilon_{E}}{\left(\frac{K_{IS}}{K_{SS}}\right)\varepsilon_{E}-\left(\frac{K_{ES}}{K_{SS}}\right)\varepsilon_{I}}, \end{array}$$

to the first order approximation. Considering a special case when the E and I inputs are elicited at the same location, then we have *K*_*ES*_ = *K*_*IS*_ = *αK*_*SS*_, where *α* is a number less than one, which reflects the effect of spatial dependence of transfer resistance. The above results can be simplified as

$$\begin{array}{c} g_{E}^{eff}=\frac{b+k\varepsilon_{I}/\alpha }{\varepsilon_{I}-\varepsilon_{E}}, \end{array}$$ (27)

$$\begin{array}{c} g_{I}^{eff}=\frac{b+k\varepsilon_{E}/\alpha }{\varepsilon_{E}-\varepsilon_{I}}. \end{array}$$ (28)

If the traditional method is used, the estimated conductance can be obtained by setting *α* = 1 in Eqs 27 and 28. Therefore, the absolute errors for the conductance determined by the traditional method are

$$\begin{array}{c} \Delta g_{E}^{eff}=\frac{k\varepsilon_{I}\left(1-\frac{1}{\alpha }\right)}{\varepsilon_{I}-\varepsilon_{E}}, \end{array}$$ (29)

$$\begin{array}{c} \Delta g_{I}^{eff}=\frac{k\varepsilon_{E}\left(1-\frac{1}{\alpha }\right)}{\varepsilon_{E}-\varepsilon_{I}}. \end{array}$$ (30)

Clearly, the ratio between the two absolute errors is $\Delta g_{E}^{eff}:\Delta g_{I}^{eff}=-\varepsilon_{I}:\varepsilon_{E}$. From Eqs 29 and 30, $\Delta g_{E}^{eff}$ and $\Delta g_{I}^{eff}$ are both negative, which indicates that the conductance measured by the traditional method is always smaller than the true effective conductance.

#### Nonexistence of a transform between the local conductance and the conductance determined by the traditional method

Here we demonstrate that the local conductance and the conductance determined by the traditional method are not related because there does not exist a transform between the local E (I) conductance on the dendrite and the E (I) conductance determined by the traditional method.

We demonstrate this by using the method of proof by contradiction. Let’s assume there are such transforms that link the conductances determined by the traditional method ($g_{E}^{tr}$ and $g_{I}^{tr}$) and the local ones (*g*_*E*_ and *g*_*I*_), i.e., $g_{E}^{tr}=F\left(g_{E}\right)$, $g_{I}^{tr}=G\left(g_{I}\right)$. On the one hand, according to the traditional method, $g_{E}^{tr}$ and $g_{I}^{tr}$ shall satisfy Eqs 2 and 3. Therefore, we have

$$\begin{array}{c} k=F\left(g_{E}\right)+G\left(g_{I}\right),\\ b=F\left(g_{E}\right)\varepsilon_{E}+G\left(g_{I}\right)\varepsilon_{I}. \end{array}$$

By expanding *F*(*g*_*E*_) and *G*(*g*_*I*_) as Taylor series to the first order accuracy, we have

$$\begin{array}{c} k=F_{0}+G_{0}+F_{1}g_{E}+G_{1}g_{I}+o\left(g\right), \end{array}$$ (31)

$$\begin{array}{c} b=F_{0}\varepsilon_{E}+G_{0}\varepsilon_{I}+F_{1}g_{E}\varepsilon_{E}+G_{1}g_{I}\varepsilon_{I}+o\left(g\right), \end{array}$$ (32)

where *F*_0_, *F*_1_, *G*_0_, and *G*_1_ are the coefficients of the Taylor series. On the other hand, from Eqs 24 and 25, we have *g*_*E*_ and *g*_*I*_ satisfying

$$\begin{array}{c} k={\left(\frac{K_{ES}}{K_{SS}}\right)}^{2}g_{E}+{\left(\frac{K_{IS}}{K_{SS}}\right)}^{2}g_{I}+o\left(g\right), \end{array}$$ (33)

$$\begin{array}{c} b=(\frac{K_{ES}}{K_{SS}})g_{E}\varepsilon_{E}+(\frac{K_{IS}}{K_{SS}})g_{I}\varepsilon_{I}+o\left(g\right). \end{array}$$ (34)

By comparing the coefficients in Eq 31 and those in Eq 33, we have

$$\begin{array}{c} F_{1}={\left(\frac{K_{ES}}{K_{SS}}\right)}^{2},\;\;\;\;G_{1}={\left(\frac{K_{IS}}{K_{SS}}\right)}^{2}. \end{array}$$ (35)

By comparing the coefficients in Eq 32 and those in Eq 34, we have

$$\begin{array}{c} F_{1}=\frac{K_{ES}}{K_{SS}},\;\;\;\;G_{1}=\frac{K_{IS}}{K_{SS}}. \end{array}$$ (36)

For the synaptic inputs received on the dendrite, we have *K*_*ES*_ ≠ *K*_*SS*_ and *K*_*IS*_ ≠ *K*_*SS*_. Therefore, Eq 35 contradicts to Eq 36, which proves that there do not exist such transforms between the local conductance and the conductance determined by the traditional method, indicating that the local conductance and the conductance determined by the traditional method may not be correlated with each other.

## Results

### Relation between effective conductance and local conductance

As introduced in the section of Materials and Methods, the effective conductance is defined by the ratio of the synaptic current $I_{syn}^{eff}$ arriving at the soma to the driving force (difference between the reversal potential *ε* and the somatic membrane potential *V*) in the presence of either E or I input, i.e.,

$$\begin{array}{c} g^{eff}=\frac{I_{syn}^{eff}}{\varepsilon -V}. \end{array}$$ (37)

It should be stressed that, in order to distinguish from the synaptic current measured using voltage clamp in the traditional method, $I_{syn}^{eff}$ is the synaptic current in the absence of any externally injected current.

**Fig 1:**
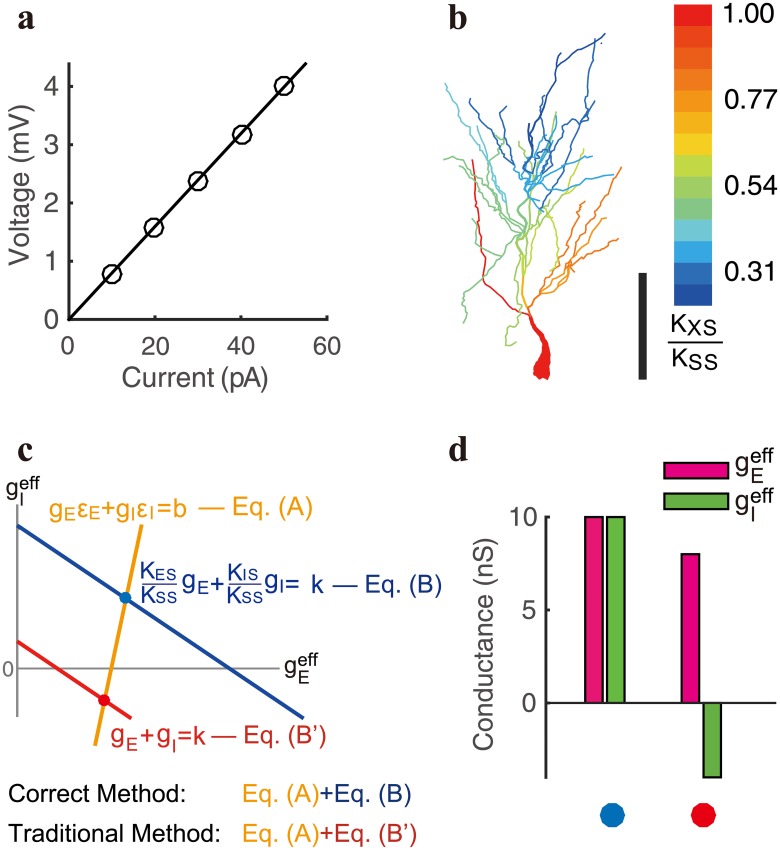
Properties of transfer resistance in the determination of effective conductance. **a**, linear dependence of somatic voltage on the injected current in our realistic neuron model. The slope is the transfer resistance between the input location and the soma. Here the current is injected on the dendrite 50 *μ*m away from the soma. **b**, spatial profile of the ratio *K*_*XS*_/*K*_*SS*_ on the dendritic arbor in stratum radiatum of the realistic neuron. *K*_*XS*_ is the transfer resistance between the input location *X* and the soma *S*, *K*_*SS*_ is the input resistance at the soma. Scalar bar indicates 100 *μ*m. **c**, an example to illustrate the deficiency of the traditional method in the determination of effective conductance. For a pair of E and I synaptic inputs on the dendrite with *K*_*ES*_ = *K*_*IS*_ = 0.2*K*_*SS*_, the corresponding effective conductance values are determined by the intersection between the orange and blue lines, i.e., solving Eq. (A) and Eq. (B). Were the traditional approach used to determine the conductance, the intersection between the orange and red lines would yield the corresponding inaccurate conductances, i.e., solving Eq. (A) and Eq. (B’). **d**, the value of the conductances determined in **c**. Colored circles on the bottom indicate the cases of the intersection points with the corresponding color in **c**. Note that for the case marked by red, the value of I effective conductance is negative via the traditional method.

By performing a static transfer resistance analysis (see Materials and methods for details), one can show that the effective conductance at the soma depends linearly on the local conductance on the dendrite,

$$\begin{array}{c} g_{E}^{eff}=\frac{K_{ES}}{K_{SS}}g_{E}\;\;\;\;\;\;\;\text{and}\;\;\;\;\;\;\;\;g_{I}^{eff}=\frac{K_{IS}}{K_{SS}}g_{I} \end{array}$$ (38)

to the first order accuracy of *g*_*E*_ and *g*_*I*_, where $g_{E}^{eff}$ and $g_{I}^{eff}$ are the effective E and I conductances respectively, and *g*_*E*_ and *g*_*I*_ are the corresponding local ones. Here the transfer resistance *K*_*AB*_ is defined as the ratio of the voltage change in location B to the magnitude of the injected current in location A. The validity of the static transfer resistance analysis and the derived linear relationship between the effective and local conductances (Eq 38) relies on the assumption that transfer resistance is a well-defined property of a neuron independent of input strength, which has been verified in the simulation of our realistic pyramidal neuron model (Fig 1a).

The effective conductance is an important concept not only because it is a proportional indicator of the local conductance on the dendrite (Eq 38), but also it reflects directly the functional impact of synaptic inputs on the spike trigger mechanism and thereby neuronal information processing at the soma. For example, a strong synaptic input at distal dendrite and a weak synaptic input at proximal dendrite can give rise to a similar magnitude of effective conductance when they arrive at the soma after dendritic filtering and integration, thus inducing a similar somatic response to initiate action potentials and propagate signals. Therefore, measuring the effective conductance is valuable for understanding the influence of synaptic activities on somatic membrane potential change and information coding.

### Deficiency of the traditional method revealed in theoretical analysis

On account that the clamp can control the voltage sufficiently well only at the soma and its nearby regions, by applying the somatic voltage clamp, it is difficult to measure the local conductance accurately. However, it remains possible to measure the effective conductance which by definition only requires the local control of the voltage at the soma. As will be demonstrated below, the conductance determined by the traditional method (using Eqs 2 and 3) is close to neither the local conductance on the dendrite nor the effective conductance at the soma.

Here we perform the static transfer resistance analysis to illustrate the deficiency of the traditional method. When a neuron receives both E and I synaptic inputs on the dendrite with its somatic membrane potential clamped at various levels, our analysis yields a linear relation between synaptic current and voltage in voltage clamp mode (see Materials and methods for details), i.e., $I_{syn}^{inj}=-kV+b$, where

$$\begin{array}{c} k={\left(\frac{K_{ES}}{K_{SS}}\right)}^{2}g_{E}+{\left(\frac{K_{IS}}{K_{SS}}\right)}^{2}g_{I} \end{array}$$ (39)

and

$$\begin{array}{c} b=\frac{K_{ES}}{K_{SS}}g_{E}\varepsilon_{E}+\frac{K_{IS}}{K_{SS}}g_{I}\varepsilon_{I} \end{array}$$ (40)

to the first order accuracy. Alternatively, if we cast Eqs 39 and 40 in terms of effective conductance using Eq 38, we can obtain

$$\begin{array}{c} k=\frac{K_{ES}}{K_{SS}}g_{E}^{eff}+\frac{K_{IS}}{K_{SS}}g_{I}^{eff} \end{array}$$ (41)

and

$$\begin{array}{c} b=g_{E}^{eff}\varepsilon_{E}+g_{I}^{eff}\varepsilon_{I} \end{array}$$ (42)

to the first order accuracy. Therefore, to determine the local conductance, one needs to solve *g*_*E*_ and *g*_*I*_ from Eqs 39 and 40; while, to determine the effective conductance, one needs to solve $g_{E}^{eff}$ and $g_{I}^{eff}$ from Eqs 41 and 42. Clearly, in contrast to Eq 2 in the traditional method, the slope *k* of the I-V relation in Eqs 39 or 41 is neither the total effective conductance nor the total local conductance. In this sense, the conductance determined by the traditional method using Eqs 2 and 3 is neither the local conductance nor the effective conductance. We can further show that there does not exist a transform between the local conductance and the conductance determined by the traditional method (see Materials and methods), indicating that the measured conductance may not correlate with the local conductance thus its biological interpretation is unclear.

The error of the traditional method is caused by the prefactors $\frac{K_{ES}}{K_{SS}}$ and $\frac{K_{IS}}{K_{SS}}$ in Eqs 39–41, which arise from the *nonlinear interaction* between the injected current at the soma and the synaptic current from the dendrite (see Materials and methods for details). Only when the E and I inputs are given at the soma will the prefactors vanish. In this particular limit, the local and effective conductances become identical (Eq 38). In addition, Eqs 39, 40, 41 and 42 further reduce to Eqs 2 and 3, which enables one to use the traditional method to determine the local or effective conductance accurately. However, in general, these prefactors cannot be naively assumed to be unity (i.e., no nonlinear interaction) since they can distort significantly the determination of conductance, as will be demonstrated below.

As it is challenging in experiment to elicit inputs which are spatially broadly distributed on the distal dendrite, we resort to numerical simulations using the realistic pyramidal neuron model to investigate the spatial dependence of the prefactors $\frac{K_{ES}}{K_{SS}}$ and $\frac{K_{IS}}{K_{SS}}$. Our numerical result shows that the prefactors across the entire dendritic tree decay from unity to zero rapidly with the increase of the distance between the synaptic input sites and the soma (Fig 1b). Therefore, if one attempts to determine the effective conductance using the traditional method based on Eqs 2 and 3 rather than Eqs 41 and 42, the errors can become prominent. For instance, when the E and I inputs are given at the distal dendrite where the prefactors are small, the value of conductance could vanish when determined by the traditional method. In addition, in our analysis, when the prefactors become sufficiently small, a negative conductance can arise via the traditional method (Fig 1c and 1d). This possibly explains why a negative conductance was observed in early experiments [26]. Our theoretical prediction is particularly of note that the measurement of I conductance is distorted more significantly than E conductance by the traditional method—as a consequence of the ratio of measurement error between the E and I conductances being proportional to the ratio between the I reversal potential *ε*_*I*_ (e.g., −10 mV relative to the resting potential) and the E reversal potential *ε*_*E*_ (e.g., 70 mV relative to the resting potential), i.e., Δ*g*_*E*_: Δ*g*_*I*_ = −*ε*_*I*_: *ε*_*E*_ (see Fig 1c and Materials and methods).

### Deficiency of the traditional method revealed in electrophysiological experiments

**Fig 2:**
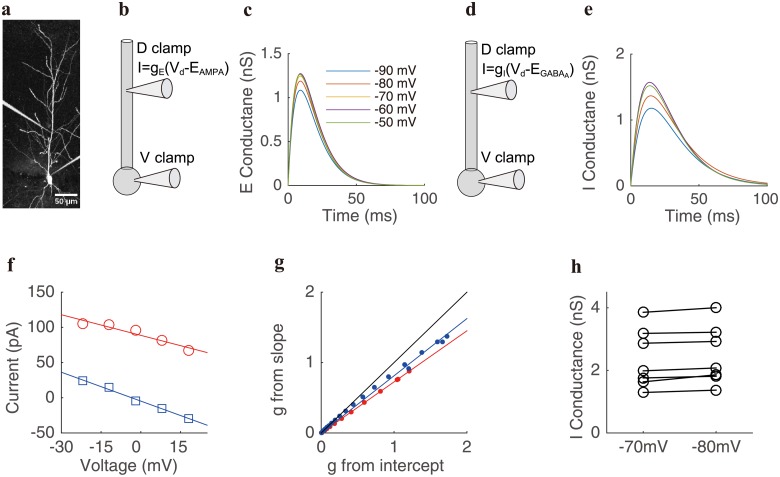
Determination of individual effective conductance in experiments. **a**, an image showing simultaneous dendritic and somatic recordings on a hippocampal CA1 pyramidal cell that was filled with fluorescence dye Alexa Fluor-488. **b**, schematic diagram of the recording configuration when a pyramidal neuron receives an individual E input. A voltage clamp is made at the soma and a dynamic clamp is made on the dendritic trunk about 100 *μ*m away from the soma. Somatic voltage is clamped from −90 mV to −50 mV when an individual E input is given by dynamic clamp on the dendrite. The local E conductance takes the form proportional to *e*^−*t*/5^ − *e*^−*t*/7.8^ (time unit: ms) with a peak amplitude of 2.5 nS. **c**, $g_{E}^{inj}$ obtained as $I_{syn}^{inj}/\left(\varepsilon_{E}-V\right)$ by least square fitting of a double exponential function. **d**, schematic diagram of the recording configuration when a pyramidal neuron receives an individual I input—same as in **b** except that the local I conductance takes the form proportional to *e*^−*t*/6^ − *e*^−*t*/18^ (time unit: ms) with a peak amplitude of 4 nS. **e**, $g_{I}^{inj}$ obtained as $I_{syn}^{inj}/\left(\varepsilon_{I}-V\right)$ by least square fitting of a double exponential function (cf. **c** for color coding). Not shown is the case for the clamp voltage at the absolute reversal potential of −70 mV (see text). **f**, I-V relations corresponding to E (red) and I (blue) inputs under the five holding voltage levels, respectively. The data are collected at the time 10 ms after the onset of the stimulus. The value of the voltage shown in the abscissa is relative to the resting potential. **g**, the relation between the conductance determined from the slope (Eq 2) and the intercept (Eq 3). The E and I conductances are marked by red and blue color, respectively. Each dot corresponds to the conductance value at one time point. The red and blue lines are corresponding linear fitting. For reference, the black reference line has a slope of unity. **h**, the independence of the I conductance value on the change of the I reversal potential. The circles on the left and right columns are the peak I conductances determined with the I reversal potential $E_{GABA_{A}}=-70\;\text{mV}$ and $E_{GABA_{A}}=-80\;\text{mV}$, respectively. The solid lines link the conductances determined at the same neuron. The mean relative change of the conductance under different reversal potentials is within 5%.

From our theoretical analysis above, the deficiency of the traditional method in measuring the effective conductance results from the neglect of the nonlinear interaction between synaptic current and injected clamp current. To confirm our theoretical results, we perform electrophysiological experiment to demonstrate the existence of the interaction between synaptic current and injected clamp current. In the experiment (see Materials and methods for details), we record 7 rat hippocampal CA1 pyramidal neurons using somatic voltage clamp. The resting potential of each neuron ranges from −57 mV to −68 mV. E and I synaptic inputs are given via a dynamic clamp at the location on the dendrite about 100 *μ*m away from the soma. The absolute E and I reversal potentials are set as *E*_*AMPA*_ = 0 mV and $E_{GABA_{A}}=-70\;\text{mV}$, and the relative reversal potentials *ε*_*E*_ and *ε*_*I*_ can be determined by subtracting the resting potential from *E*_*AMPA*_ and $E_{GABA_{A}}$ respectively. The local input synaptic conductances through the dynamic clamp take the form of a difference of two exponential functions whose time constants were derived from voltage traces in experiment [28], with rise time constant 5 ms (6 ms) and decay time constant 7.8 ms (18ms) for E (I) conductance. The peak amplitude of E and I synaptic conductances ranges from 2 nS to 5 nS and 3 nS to 6 nS, respectively. For each pyramidal neuron, we clamp the voltage at the soma with five levels from −50 mV to −90 mV with an increment of 10 mV (Fig 2a). For an individual E input given on the dendrite via dynamic clamp (Fig 2b), we can then record five excitatory postsynaptic current (EPSC) traces $I_{syn}^{inj}$ at the soma corresponding to the five holding voltage levels, and determine the corresponding E conductance traces using $g_{E}^{inj}=I_{syn}^{inj}/\left(\varepsilon_{E}-V\right)$. Here *ε*_*E*_ and *V* are reversal and holding potentials relative to the resting potential, and $I_{syn}^{inj}$ is the synaptic current which is the current increment from the baseline injected current that is used to clamp the neuron to a steady state of voltage (see Materials and methods). Again, the superscript “*inj*” in the notations $g_{E}^{inj}$ and $I_{syn}^{inj}$ emphasizes the fact that they are determined *in the presence of* the injected clamp current. We obtain the final profile of the resulting conductance as a function of a difference of two exponentials using least square fitting. Fig 2c shows that five E conductances $g_{E}^{inj}$ thus obtained are not identical with disparity between these conductances well beyond recording statistical fluctuations. The dependence of the conductance value of $g_{E}^{inj}$ on the clamp voltage as shown in Fig 2c contradicts the assumption of the traditional method that the synaptic conductance is independent of injected current as described in Eq 1. It confirms our result that the synaptic current from the dendrite and the injected current on the soma cannot be linearly summed as a consequence of their interaction with each other. The difference between the I conductances $g_{I}^{inj}$ estimated from different voltage clamp levels is more prominent than the E case (Fig 2d and 2e). The voltage dependence of the I conductance $g_{I}^{inj}$ can be highlighted by the following limiting case. When the soma is clamped at the I reversal potential $E_{GABA_{A}}=-70\;\text{mV}$, the value of the I conductance $g_{I}^{inj}$ would become unphysically infinity because the denominator in the expression $g_{I}^{inj}=I_{syn}^{inj}/\left(\varepsilon_{I}-V\right)$ vanishes (such a case is not displayed in Fig 2e).

There is further evidence demonstrating the interaction between synaptic current and injected clamp current as observed in the experiment. Were the synaptic and injected currents linearly summable (Eq 1), then the conductances obtained from the slope and those from the intercept of the I-V relation would be identical through the traditional method. However, this turns out not to be the case in our experimental observation. To be specific, given an individual E or I input on the dendrite, we can obtain a linear I-V relationship between the voltage and the synaptic current $I_{syn}^{inj}$ at each time point after the onset of the stimulus. An example of the I-V relationship at the time 10 ms after the stimulus onset is shown in Fig 2f. Upon casting *g*(*ε* − *V*) as $I_{syn}^{inj}$ and following Eqs 2 and 3 for the case of only a purely E or I input, we obtain the ratio of the conductance value estimated from the slope to that from the intercept. This ratio deviates greatly from unity—the expected result obtained by the traditional method. It is nearly a constant and is independent of conductance amplitude (Fig 2g). The value of ratio for E inputs is nearly identical to that for I inputs when the E and I inputs are given at the same dendritic location (Fig 2g). This observation is consistent with our theoretical prediction from Eqs 41 and 42 for the case of a purely E or I input, for which the above ratio is the same as the ratio of the effective conductance $g_{E}^{eff}$ ($g_{I}^{eff}$) at the soma to the local conductance *g*_*E*_ (*g*_*I*_) on the dendrite (Eq 38).

### The intercept method for the determination of effective conductance

In principle, the deficiency of the traditional method could be eliminated by measuring the value of the prefactors associated with input locations based on Eqs 41 and 42. However, for a neuron receiving a large number of spatially broadly distributed synaptic inputs, it remains difficult to have all *a priori* information about the transfer resistances between the synaptic input sites and the soma, thus hampering the recovery of the E and I conductances from Eqs 41 and 42 directly. From our theoretical analysis, we note that the intercept *b* in Eq 42 possesses the form of effective reversal current without the explicit information of transfer resistances (see Materials and methods). Therefore, we propose to recover the effective E and I conductances only from the intercept value. In principle, the effective E and I conductances can be recovered from multiple I-V relations by varying the E or I reversal potential at various levels, or by pharmacologically blocking the E or I synaptic receptor. As a proof of concept, below we examplify the recovery of the effective conductances from the intercept information via the change of synaptic reversal potential. To be specific, we can vary the I reversal potential from *ε*_*I*_ to $\varepsilon_{I}^{{}^{\prime }}$ to obtain a second intercept equation

$$\begin{array}{c} b^{{}^{\prime }}=g_{E}^{eff}\varepsilon_{E}+g_{I}^{eff}\varepsilon_{I}^{{}^{\prime }}, \end{array}$$ (43)

and then the effective E and I conductances can be obtained from Eqs 42 and 43. In physiological experiment, to change reversal potential, one may need to effect a change of the intracellular fluid environment as further discussed in the section of Discussion. From now on, we refer to this method based on Eqs 42 and 43 as the intercept method (IM), and the traditional method based on Eqs 2 and 3 as the slope-and-intercept method (SIM).

The key difference between SIM and IM lies in the transfer resistance. Based on our static transfer resistance analysis, the slope of the linear I-V relation follows Eq 41, and the intercept of the linear I-V relation follows Eq 42, in which the pre-factors *K*_*ES*_/*K*_*SS*_ and *K*_*IS*_/*K*_*SS*_ are determined by the synaptic input locations, and their values are in general difficult to measure.

Conceptually, IM does not ignore these pre-factors but SIM does. In IM, by accounting for the fact that these pre-factors are generally unknown, one can only use the intercept information to recover $g_{E}^{eff}$ and $g_{I}^{eff}$. And because there are two unknown variables $g_{E}^{eff}$ and $g_{I}^{eff}$ to solve, IM suggests to provide at least two equations of I-V relations. For example, if the reversal potential is changed, one can obtain a second intercept equation (Eq 43). Subsequently, in IM, the effective E and I conductances $g_{E}^{eff}$ and $g_{I}^{eff}$ are determined by solving Eqs 42 and 43.

In contrast, in SIM, by naively assuming the location-dependent pre-factors in Eq 41 to be unity, the effective E and I conductances are determined by solving Eqs 41 and 42 (with the assumption that *K*_*ES*_/*K*_*SS*_ = 1 and *K*_*IS*_/*K*_*SS*_ = 1 in Eq 41). However, based on the static transfer resistance analysis, the existence of the pre-factors results from the nonlinear interaction between the clamp current at the soma and the synaptic current from the dendrites, and the pre-factors in general deviate from unity as shown in Fig 1b. Therefore, the conductances measured by the traditional method is expected to deviate from the true effective conductances, as shown in both electrophysiological experiments and realistic neuron simulations below.

Technically, when applying the intercept method to measure the effective E and I synaptic conductances of a neuron, the first step is to clamp the somatic voltage at various levels and measure the corresponding synaptic currents arriving at the soma. In this step, one shall exert a well control of the experimental condition such that the E and I synaptic inputs received by the neuron under different holding voltage are approximately the same. This step is identical to that in the traditional slope-and-intercept method. The second step is to fit a linear relation between the holding voltage and the synaptic current and read out the intercept value from the I-V relation at each time point, which contains information of the effective E and I conductances $g_{E}^{eff}$ and $g_{I}^{eff}$ described by Eq 42. The third step is to vary the reversal potential to a different value and repeat the first two steps to obtain its corresponding intercept value at each time point, which also contains information of $g_{E}^{eff}$ and $g_{I}^{eff}$ described by Eq 43. The final step is to recover $g_{E}^{eff}$ and $g_{I}^{eff}$ by solving Eqs 42 and 43.

### Validation of the intercept method in electrophysiological experiments

We next perform experiment to demonstrate the validity of IM by contrasting its error with that of SIM. Because we need to vary the reversal potential to a different value in IM, we have first verified that the value of the effective conductance is nearly independent of the reversal potential value (Fig 2h).

Next, we proceed to determine the reference effective E and I conductances. Based on our analysis (Eq 42), the conductance obtained from the intercept of the I-V relation is the true effective conductance, i.e., the conductance at the soma induced by a synaptic input on the dendrite *in the absence of* the injected current. Therefore, we choose the values of the E and I conductances estimated from the intercepts for the case of only pure E or I inputs as the *reference conductances* to evaluate IM and SIM. We note in passing that the effective reference conductances determined in this way are more accurate than those determined directly from Eq 1 in the absence of voltage clamp for we can avoid taking time derivative of noisy experimental voltage data—a procedure that would introduce large numerical errors. In our realistic neuron simulation to corroborate our experimental results below, however, we can use Eq 1 in the absence of voltage clamp to determine the reference conductance since the numerical simulation is sufficiently accurate for obtaining the time derivative of voltage.

**Fig 3:**
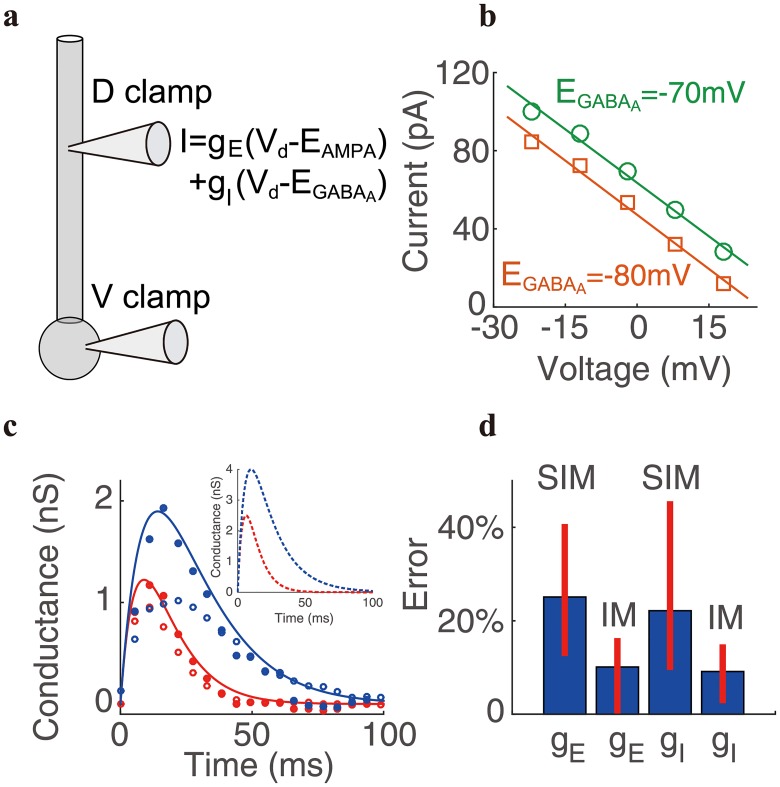
Determination of a pair of effective conductances in experiment. **a**, schematic diagram of the recording configuration when a pyramidal neuron receives a pair of E and I inputs. A voltage clamp is made at the soma and a dynamic clamp is made on the dendritic trunk about 100 *μ*m away from the soma. Somatic voltage is clamped from −90 mV to −50 mV when both E and I inputs are given simultaneously by the dynamic clamp on the dendrite. **b**, I-V relations obtained after applying the voltage clamp with the I reversal potentials $E_{GABA_{A}}=-70\;\text{mV}$ (green) and $E_{GABA_{A}}=-80\;\text{mV}$ (orange), respectively. The data are collected at the time 10 ms after the onset of the stimulus. The voltage value shown in the abscissa is relative to the resting potential. **c**, a pair of E (red) and I (blue) conductances determined by SIM (open circle) and IM (solid circle). Solid curves are the reference conductances measured by given an individual E or I input separately. Dash curves are the local E and I conductances on the dendritic trunk 100 *μ*m away from the soma. The local E conductance takes the form proportional to *e*^−*t*/5^ − *e*^−*t*/7.8^ (time unit: ms) with a peak amplitude of 2.5 nS, and the local I conductance takes the form proportional to *e*^−*t*/6^ − *e*^−*t*/18^ (time unit: ms) with a peak amplitude of 4 nS. **d**, the peak-amplitude relative error of the E and I conductances determined by IM and SIM across 7 neurons. The thick blue bar indicates the mean value of the peak amplitude error and the thin red bar indicates the range of the error.

Given an individual E pulse input at a dendritic location about 100 *μ*m away from the soma and placing the voltage clamp at the soma, we can record a set of synaptic current $I_{syn}^{inj}$ under five holding voltages from −50 mV to −90 mV. We then determine the effective E conductance from the intercept of the I-V relation at each time point. A similar procedure is carried out separately for the effective I conductance. A pair of measured effective E and I conductances determined in this way is displayed in Fig 3c as the reference values (solid curves), against which we evaluate the performance of IM and SIM.

Next, with a voltage clamp placed at the soma, we simultaneously elicit the E and I pulse inputs same as for the reference ones, i.e., input at the same dendritic location with the same strength (Fig 3a). Five total synaptic currents $I_{syn}^{inj}$ at the soma are obtained under five holding voltages from −50 mV to −90 mV. We then also observe a linear relation between the synaptic current and the membrane potential at each time point. An example of the I-V relation at the time 10 ms after the onset of the stimulus is shown in Fig 3b. Finally, we determine a pair of values of the E and I conductance pulses from the linear I-V relation by using SIM. Meanwhile, by changing the I reversal potential $E_{GABA_{A}}$ from −70 mV to −80 mV and repeating the above procedure (Fig 3b), a pair of alternative values of E and I conductances can be obtained using IM. By comparing the values of the conductance pulses measured by the two methods with those of the reference conductance pulses in Fig 3c, we observe that the conductance estimated by IM nearly overlaps with the true conductance, whereas the conductance estimated by the traditional SIM deviates greatly from the true conductance. As shown in Fig 3d, across 7 pyramidal neurons, the effective conductance measured by IM has a relatively small error on average, with a relative error of peak amplitude ranging from 0.6% to 15.6% for E conductance and from 3.0% to 14.3% for I conductance. In contrast, the conductance measured by SIM yields a large relative error of peak amplitude as great as from 13.1% to 40.0% for E conductance and from 10.1% to 44.9% for I conductance. According to our theoretical analysis, the error in SIM is caused by failing in taking into account the nonlinear interaction between the synaptic current from the dendrite and the injected current at the soma—thus missing the prefactors *K*_*ES*_/*K*_*SS*_ and *K*_*IS*_/*K*_*SS*_ in Eq 41, whose strength has a sensitive dependence on the dendritic location (Fig 1b). As observed in a previous experiment [26], for synaptic inputs received at the proximal dendrite 100 *μ*m away from the soma, the recovered synaptic current at the soma by using the somatic voltage clamp is already below 60%, and the escape voltage level is above 50%, indicating that *K*_*ES*_/*K*_*SS*_ and *K*_*IS*_/*K*_*SS*_ in Eq 41 are unneglectable in this case. In our experiment, we show that when the inputs are given at the proximal dendrite about 100 *μ*m away from soma, the error of SIM has already reached ∼ 40% (Fig 3d). For a synaptic input location further towards the distal dendrite, the error is expected to become substantially larger.

### Validation of the intercept method in realistic neuron simulations

As it is a rather challenging experimental task to elicit inputs on multiple dendritic locations in general, and on the distal dendrite in particular, we turn to realistic neuron simulations to demonstrate the validity of IM by contrasting its error with that of SIM for distal inputs or spatiotemporally broadly distributed multiple inputs on the dendrite. In our realistic pyramidal neuron model (see Materials and methods for details), the resting potential is set to *V*_*r*_ = −70 mV, and the absolute E and I reversal potentials are initially set to *E*_*AMPA*_ = 0 mV, $E_{GABA_{A}}=-80\;\text{mV}$. The relative reversal potentials are then determined as *ε*_*E*_ = 70 mV and *ε*_*I*_ = −10 mV.

**Fig 4:**
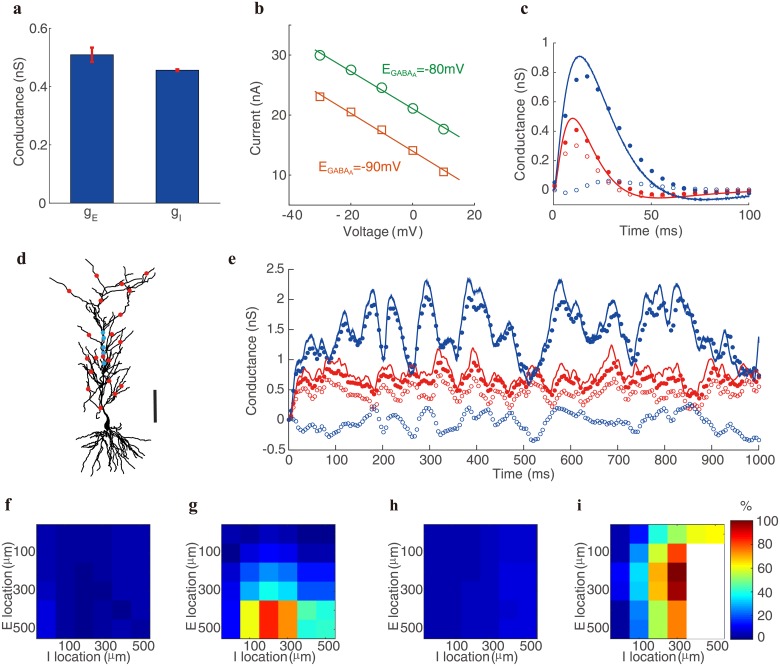
Determination of effective conductances in realistic neuron simulations. **a**, independence of the E and I conductances on the change of the reversal potentials. The E reversal potential *E*_*AMPA*_ varies from −50 mV to 50 mV with ten even increments, and the I reversal potential $E_{GABA_{A}}$ varies from −70 mV to −90 mV with five even increments. The thick blue bar indicates the mean and the thin red bar indicates one standard deviation. The ratio of standard deviation to mean for both conductance values is within 5%. **b**, Two I-V relations obtained under somatic voltage clamp mode with the I reversal potential $E_{GABA_{A}}$ changing from −80 mV (green) to −90 mV (orange). The data are collected at the time 15 ms after the onset of the stimulus. The voltage value shown in the abscissa is relative to the resting potential. **c**, a pair of E (red) and I (blue) effective conductances determined by SIM (open circle) and IM (solid circle). The E and I inputs are given simultaneously on the dendritic trunk about 350 *μ*m and 300 *μ*m away from the soma, respectively. Solid curves are the reference conductances obtained under the same individual E or I input given separately. **d**, the spatial distribution of multiple E (red) and I (blue) inputs on the dendrite. Bar indicates 100 *μ*m. **e**, E (red) and I (blue) effective conductances determined by SIM (open circle) and IM (solid circle) when the neuron receives multiple inputs. Solid curves are the reference conductances obtained by the same E or I inputs given separately. The input locations are shown in **d** and the input times at each location are uniformly distributed from 0 ms to 1000 ms with a rate of 100 Hz. **f-i**, spatial dependence of the relative error for the E and I conductance measurement. Here, the locations for a pair of E and I input of constant conductances are scanned across the dendrite. The location distance is measured from the soma. **f-g** are the error of E conductance measured by IM and SIM, respectively, and **h-i** are the error of I conductance measured by IM and SIM, respectively. They share a color bar to indicate the percentage of error. In **i**, the large white area, which corresponds to negative conductance values, again illustrates the failure of the traditional method.

We first demonstrate the validity of our realistic neuron model as a good model of a biological neuron by examining whether the performance of SIM and IM for the model neuron is similar to their performance for the pyramidal neurons recorded in the experiment, when the E and I synaptic inputs are given at the dendritic trunk of the model neuron about 100 *μ*m away from the soma—the same input condition as that in the experiment. In the model, we measure the distance by taking into account the zig-zag geometry of the dendrites. As above, we first determine the reference E and I conductances by giving the neuron an individual E and I input separately. For an individual E pulse input at a dendritic location about 100 *μ*m away from the soma but without the injected clamp current at the soma, we can numerically record the corresponding EPSP at the soma and invoke Eq 37 to determine the value of the effective E conductance pulse from the point-neuron model (for which we set *I*_*inj*_ = 0, *g*_*I*_ = 0 in Eq 1). A similar procedure can be carried out for the effective I conductance pulse in response to an individual I pulse input at a dendritic location about 100 *μ*m away from the soma (again, in the absence of injected current at the soma). In the simulation, the experimental result is also confirmed that the value of the effective E or I conductance is nearly identical under different synaptic reversal potentials (Fig 4a). When the E and I inputs are given simultaneously, the application of voltage clamp gives rise to an I-V relation at each time point as in experiment. By altering the I reversal potential $E_{GABA_{A}}$ from −80 mV to −90 mV, an additional I-V relation at the same time point can be obtained. As shown in S3 Fig, the effective E and I conductances measured by IM has a relative error of peak amplitude about 13.5% for E conductance and 9.6% for I conductance. In contrast, the conductance measured by SIM yields a large relative error of peak amplitude as great as 18.8% for E conductance and 37.4% for I conductance. This result falls within the range of the error measured in the experiment as shown in Fig 3d, and is similar to the error measured in the example neuron as shown in Fig 3c.

We next investigate the performance of IM when a pair of synaptic inputs are given on the distal dendrite of the model neuron. For simultaneous E and I inputs given at the dendritic trunk about 350 *μ*m and 300 *μ*m away from the soma respectively, the application of voltage clamp gives rise to an I-V relation at each time point as in experiment. An additional I-V relation at the same time point after the onset of the stimulus results from altering the I reversal potential $E_{GABA_{A}}$ from −80 mV to −90 mV (Fig 4b). As shown in Fig 4c, the conductances measured using IM have a small relative error compared with the corresponding reference values, with a maximum error of 14.5% for E conductance and 11.1% for I conductance in the peak amplitude. In contrast, those determined using SIM yield an error as large as 36.8% for E conductance and 98.1% for I conductance.

To model the situation *in vivo*, we distribute 15 E inputs and 5 I inputs across the entire dendritic tree of the pyramidal neuron (Fig 4d). At each synaptic location, the arrival time of each input is randomly selected between 0 ms and 1000 ms with input rate of 100 Hz. We use the identical input for both the measurement of time evolution of the effective conductance as reference without the voltage clamp (using Eq 1) as that of the conductances with the voltage clamp. Comparison of the values of conductance measured by the IM and SIM methods with the reference conductance in Fig 4e demonstrates that the effective E or I conductance estimated by our IM is in good agreement with the true effective conductance. Meanwhile, the conductance estimated by SIM deviates greatly from the true one in general, and particularly substantial for the inhibitory case. In the subthreshold regime, the conductances measured by IM incur a relatively small error with time averaged relative error of 16.3% for E conductance and 6.3% for I conductance, whereas those determined using SIM yield a time averaged relative error as large as 42.7% for E conductance and 102.6% for I conductance. In this simulation, while the true I conductance is substantially larger than the true E conductance, the I conductance estimated by SIM turns out to be substantially smaller than the E conductance. It is important to stress that the value of the I conductance could even become negative, thus demonstrating the severe deficiency of SIM. We note that in Fig 4c the tails of the reference conductances also become slightly negative, which arises from the repolarization of the membrane potential before relaxing to its resting state. This phenomenon has also been observed in experiments [28] and is attributed to the activity of voltage-gated ion channels, which have not been taken into account in the simple point neuron model (Eq 1). Different from the case in Fig 4c, the negative conductance determined by SIM in Fig 4e originates from the deficiency of the method itself instead of active channels. In fact, the negative value of conductance in Fig 4e is only observed for the inhibitory one, for which its reference conductance always stays at a positive level.

We now address the question of how the error of the two methods depends on the input locations on the dendrite. For a pair of synaptic inputs at various locations on the dendrite, our simulation shows that IM can control the error about 10% even for the distal inputs (Fig 4f and 4h), whereas the error of SIM increases rapidly to 100% as the input location moves away from the soma to the distal dendrite (Fig 4g and 4i). In some remote distal dendritic sites—greater than 400 *μ*m away from the soma, the estimated I conductance can also become negative (Fig 4i), accentuating the SIM deficiency.

To investigate the performance of SIM and IM when the inputs are given on a dendritic branch, we have simulated the following three cases, i.e., when both the E and I inputs are located at the dendritic trunk 200 *μ*m away from the soma, when the E input is located at a dendritic branch 200 *μ*m away from the soma and the I input is located at the dendritic trunk also 200 *μ*m away from the soma, and when both the E and I inputs are located at a dendritic branch 200 *μ*m away from the soma. As shown in S4 Fig, the performance of IM is almost the same for the three cases, while the performance of SIM improves a little when both the E and I inputs are located at the dendritic branch. As discussed previously, the error of SIM results from the incorrect assumption *K*_*ES*_/*K*_*SS*_ = 1 and *K*_*IS*_/*K*_*SS*_ = 1 in Eq 41. Therefore, the slightly improved performance of SIM for inputs received on secondary dendrites can be explained by the fact that the prefactors *K*_*ES*_/*K*_*SS*_ and *K*_*IS*_/*K*_*SS*_ are closer to unity when the inputs are on a branch compared to the case when the inputs are on the dendritic trunk, as shown in Fig 1b and Eqs 29 and 30.

**Fig 5:**
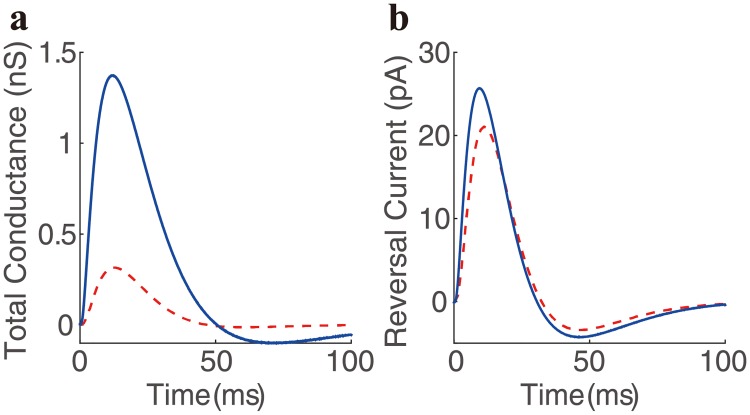
Direct validation of IM from realistic neuron simulations. Given the same pair of the synaptic inputs simultaneously as shown in Fig 4c to obtain reference effective E and I conductances $g_{E}^{eff}$ and $g_{I}^{eff}$, an I-V relation at a particular moment can be obtained by holding the somatic voltage at different levels. **a**, the deviation of the slope of the I-V relation (red dash curve) from the total conductance (blue curve) calculated as $g_{E}^{eff}+g_{I}^{eff}$. **b**, good agreement between the intercept of the I-V relation (red dash curve) and the reversal current (blue curve) calculated as $g_{E}^{eff}\varepsilon_{E}+g_{I}^{eff}\varepsilon_{I}$.

A further validation of IM is shown in Fig 5. For the same pair of transient E and I inputs in Fig 4c, on the one hand, we can measure the slope and the intercept of the I-V relation using voltage clamp when the E and I inputs are given simultaneously; on the other hand, we can determine the effective E and I reference conductances $g_{E}^{eff}$ and $g_{I}^{eff}$ when the E and I inputs are given separately and then reconstruct the total conductance as $g_{E}^{eff}+g_{I}^{eff}$ and the effective reversal current as $g_{E}^{eff}\varepsilon_{E}+g_{I}^{eff}\varepsilon_{I}$. Fig 5 shows that the effective reversal current overlaps well with the intercept of the I-V relation, while the violation of SIM is instantiated by a rather substantial difference between the total effective conductance and the slope of the I-V relation.

## Discussion

To extract biologically interpretable E and I conductances, in the present work, we have proposed the concept of the effective conductance by viewing the soma rather than the entire neuron as a uniform point (Eq 1) so as to deploy a well clamped voltage only at the soma in the presence of the space clamp effect. The effective conductance reflects intrinsically the effect of active ion channels along the dendrite and the filtering property of the dendrite on the conductance input to the soma. Furthermore, the effective conductance is a functionally important quantity because it is strongly correlated to the local postsynaptic conductance at the dendrite, and it can gauge directly the functional impact of synaptic inputs on the subthreshold dynamics and the spike trigger mechanism at the soma.

We then provide a new method—the intercept method—to measure the effective conductance accurately and demonstrate that IM produces rather good measurement of conductance values using rat hippocampal CA1 pyramidal neurons and a biologically realistic model neuron. IM can be applied in many settings in neurophysiological studies involving the E-I interaction, for instance, to understand synaptic mechanisms of sensory processing, the origin of neuronal oscillations, and the balanced nature of excitation and inhibition. From our theoretical analysis, IM is applicable for a large class of neurons in a wide physiological regime for which an approximate linear input-output relation holds [50, 51]. Incidentally, we note that IM can also be applied under a current clamp [52, 53].

In both our experiments and simulations, the synaptic time constants are derived from the voltage traces recorded in our previous experiment [28]. Despite the fact that they are relatively slow compared with those measured *in vivo*, the accuracy of our method is insensitive to the value of these parameters, which can be demonstrated in our theoretical analysis and realistic neuronal simulations. To illustrate this, we have evaluated the performance of IM and SIM in our realistic neuronal simulations when the inputs have relatively fast synaptic dynamics. Based on Ref. [26], we set the rise time constant as 0.5 ms and decay time constant as 5 ms for an individual E conductance, and the rise time constant as 1 ms and decay time constant as 10 ms for an individual I conductance. As shown in S5 Fig, IM is also effective while SIM remains to induce a large error in this case. In principle, the asymptotic analysis of the cable equation could be performed with spatiotemporal synaptic inputs, thus it is expected to generalize our theoretical results derived from the static transfer resistance analysis to the case where the temporal dynamics of synaptic inputs are incorporated. In this case, the structure of the slope and intercept of the I-V relation is expected to be similar to Eqs 41 and 42, and the prefactors may be more complicated to involve temporal convolution rather than being constant. This may help explain our simulation result showing that the outperformance of IM to SIM is insensitive to time constants of synaptic inputs.

### Comparison with previous studies

The space clamp effect has been well known for decades [21–27] which limits the control of the voltage clamp on the membrane potential across the entire dendritic arbor, thus potentially impeding the quantitative understanding of synaptic physiology, especially the interaction between excitation and inhibition. The error of conductance measurement induced by the space clamp effect has been quantified in experiment [26]. In addition, early experiments show that the leak conductance of a neuron can be blocked pharmacologically to increase membrane resistance [38], and the time course of synaptic currents can be slowed down in room temperature to increase neuronal input resistance [54]. Based on these experiments, to alleviate the space clamp issue, several approaches have been proposed including pharmacologically reducing the leak conductances of the neuron or slowing the rate of synaptic conductance changes by cooling the neuron [26, 55]. However, it has been shown in experiment that these approaches are unable to resolve the space clamp problem in real neurons [26, 55]. In particular, it has been reported in [26] that the intracellular diffusion of cesium from somatic and dendritic patch pipettes for the pharmacological reduction of resting and leak conductance improves little in the measurement of synaptic currents by the somatic voltage clamp. Alternative approaches including the voltage jump method [56, 57], dendritic recording [58], and multi-compartment neuron modeling [59] have been applied to investigate synaptic physiology at dendrites. But these approaches are unable to separate the E and I inputs information received by a real neuron, hampering the understanding of the interaction between E and I inputs.

Different from the previous works, our work is not a re-examination of the space clamp effect. We aim to develop a method to measure a biologically interpretable quantity associated with E and I synaptic inputs under the constraint of the space clamp effect. We have first addressed the relation between the measured conductance by the traditional method SIM and the local conductance in the presence of the space clamp effect. Although it has been demonstrated that the measured conductance substantially deviates from the local conductance, it remains unclear whether the conductance measured using the traditional method can encode the information of the local conductance. Our work has demonstrated that there does not exist a transform between the measured conductance and the local conductance, i.e., the measured conductance may not correlate with the local conductance. Consequently, severe problems have been revealed by our realistic neuron simulations that, by using the traditional method SIM, the excitation could be misinterpreted as being substantially larger than the inhibition, which completely contradicts to the fact that inhibition is dominant to excitation (Fig 4e). And the measured inhibitory conductance could be unphysically negative (Fig 4e). In addition, we have pointed out that the major deficiency of the traditional method SIM arises from the slope equation (Eq 2) but not the intercept equation (Eq 3), which has not been noticed by previous works and our work potentially provides some guidance for future voltage clamp data analysis. Furthermore, previous works have only investigated the error induced by the space clamp effect *per se* without providing a solution to extract E and I inputs information under the constraint of the space clamp effect. In contrast, here we have developed a method named IM to measure the effective conductance based on the intercept equation (Eq 3). The notion of the effective conductance obviates the issue of the space clamp effect since one only needs to control the clamped voltage well at the soma in our method. In addition to the space clamp effect, dendritic spines also present great challenge for the estimation of local conductance because of the high spine neck resistance [60]. However, this issue is circumvented as well if one concentrates on the influence of synaptic inputs on the soma after dendritic filtering.

### Limitations of the intercept method

Similar to several other multi-trial methods of conductance measurement [11, 13, 61, 62], a limitation of our method is the requirement of repeatable network behavior from trial to trial. To overcome this limitation, several methods have been proposed to be performed on a single voltage trace [63–67]. However, these methods are limited to special cases so far. For example, some of the single-trace methods require assumptions of the form of conductance dynamics [63] or membrane potential dynamics [65, 67]. In these cases, our method will be more efficient than them when the experiment is relatively well controlled and repeatable with a good precision. In practice, our method and the single-trace methods may complement each other depending on experimental conditions.

It is worthwhile to comment that so far our method is developed to measure the effective conductance at the soma rather than the local conductance on dendrites. In order to study the integration of synaptic inputs on local dendrites and dendritic phenomena such as dendritic spikes [68, 69], the IM method shall be further improved to infer the local conductance from the effective conductance based on our derived proportional relation between the local and effective conductance. In addition, our analysis is accurate only to the first order approximation of the effective conductance, while higher order corrections may also contribute to the conductance value; And our analysis is based on a simple point model of the soma. Yet the dendritic integration of synaptic inputs can potentially lead to a more complicated form of a point-neuron model of the soma [70];

Further, the change of reversal potential in our method in principle requires a change of the intracellular fluid environment, which could be experimentally challenging especially *in vivo*. Therefore, our method is a proof of concept at the current stage, but the concept of IM already contrasts the severe deficiency of the traditional method. In addition, to provide more potential solutions, we have proposed a new alternative method to obtain a second intercept equation, i.e., intracellular blockade of GABA receptors using drugs. For example, it has been shown in experiment that fluoride ions were effective for intracellular blockade of IPSCs [71], which may make IM effective potentially. However, this approach so far has additional cellular effects as reducing a neuron’s selectivity and utility [71]. Therefore, despite that a direct validation of IM is difficult to achieve at present, we believe that our method will become useful with the further development of pharmacological tools. To move further steps, it is important to address these issues in future studies for a quantitative understanding of the synaptic dynamics of neurons with higher accuracy.

## Supporting information

S1 Fig Quantification of the distance-dependent loss of recovered synaptic current and dendritic voltage control when synaptic inputs were generated from apical dendritic sites. Recovered current (*I*_recovered_) represents percentage of injected current, and dendritic voltage escape (*V*_escape_) represents percentage of the amplitude of EPSPs recorded under current clamp at the dendritic site of generation. Data are generated from our realistic neuron simulation.
(EPS)

S2 Fig The matrix of *K*_*XY*_ normalized by *K*_*SS*_ measured from realistic neuron simulations. *K*_*XY*_ is measured as the ratio of the voltage response recorded at location *Y* to the amplitude of the injecting current at location *X* on the dendrite. The distance is measured from the soma. The symmetry of the matrix with respect to its diagonal line indicates that *K*_*XY*_ = *K*_*YX*_.
(EPS)

S3 Fig Determination of a pair of effective conductances in realistic neuron simulations. **a**, Diagram of the recording configuration when the pyramidal neuron model receives a pair of E and I inputs on the dendritic trunk about 100 *μ*m away from the soma. A voltage clamp is made at the soma. **b**, a pair of E (red) and I (blue) conductances determined by SIM (open circle) and IM (solid circle). Solid curves are the reference conductances measured by given an individual E or I input separately.
(EPS)

S4 Fig E (red) and I (blue) effective conductances determined by SIM (open circle) and IM (solid circle) when synaptic inputs are received on the dendritic trunk or on a dendritic branch. Solid curves are the reference conductances obtained by the same E or I inputs given separately. **a**, the case when both the E and I inputs are located at the dendritic trunk 200 *μ*m away from the soma. **b**, the case when the E input is located at a dendritic branch 200 *μ*m away from the soma and the I input is located at the dendritic trunk also 200 *μ*m away from the soma. **c**, the case when both the E and I inputs are located at a dendritic branch 200 *μ*m away from the soma. In each figure, the red dot on the dendritic tree is the E input location and the blue dot on the dendritic tree is the I input location.
(EPS)

S5 Fig E (red) and I (blue) effective conductances determined by SIM (open circle) and IM (solid circle) when the neuron receives multiple inputs with relatively fast synaptic dynamics. Solid curves are the reference conductances obtained by the same E or I inputs given separately. The input locations are shown in Fig 4d and the input times at each location are uniformly distributed from 0 ms to 1000 ms with a rate of 100 Hz. Each local E conductance takes the form proportional to *e*^−*t*/0.5^ − *e*^−*t*/5^ and each I conductance takes the form proportional to *e*^−*t*/1^ − *e*^−*t*/10^ (time unit: ms). The time-averaged error of SIM for E conductance is 37.0%, and for I conductance is 110.1%. In contrast, the time-averaged error of IM for E conductance is 11.7%, and for I conductance is 4.6%.
(EPS)

S1 Data The experiment data of a sample neuron. (ZIP)

## References

[1] Mariño J, Schummers J, Lyon DC, Schwabe L, Beck O, Wiesing P, et al Invariant computations in local cortical networks with balanced excitation and inhibition. Nature Neuroscience. 2005;8(2):195. 10.1038/nn1391 15665876

[2] Yizhar O, Fenno LE, Prigge M, Schneider F, Davidson TJ, O’Shea DJ, et al Neocortical excitation/inhibition balance in information processing and social dysfunction. Nature. 2011;477(7363):171–178. 10.1038/nature10360 21796121 PMC4155501

[3] Denève S, Machens CK. Efficient codes and balanced networks. Nature neuroscience. 2016;19(3):375 10.1038/nn.4243 26906504

[4] Wehr M, Zador AM. Balanced inhibition underlies tuning and sharpens spike timing in auditory cortex. Nature. 2003;426(6965):442–446. 10.1038/nature02116 14647382

[5] Zerlaut Y, Destexhe A. Enhanced responsiveness and low-level awareness in stochastic network states. Neuron. 2017;94(5):1002–1009. 10.1016/j.neuron.2017.04.001 28595044

[6] Poleg-Polsky A, Diamond JS. Retinal circuitry balances contrast tuning of excitation and inhibition to enable reliable computation of direction selectivity. Journal of Neuroscience. 2016;36(21):5861–5876. 10.1523/JNEUROSCI.4013-15.2016 27225774 PMC4879202

[7] Sprekeler H. Functional consequences of inhibitory plasticity: homeostasis, the excitation-inhibition balance and beyond. Current Opinion in Neurobiology. 2017;43:198–203. 10.1016/j.conb.2017.03.014 28500933

[8] Buzsáki G, Wang XJ. Mechanisms of gamma oscillations. Annual review of neuroscience. 2012;35:203 10.1146/annurev-neuro-062111-150444 22443509 PMC4049541

[9] van Vreeswijk C, Sompolinsky H. Chaos in neuronal networks with balanced excitatory and inhibitory activity. Science. 1996;274(5293):1724–1726. 10.1126/science.274.5293.1724 8939866

[10] Dehghani N, Peyrache A, Telenczuk B, Le Van Quyen M, Halgren E, Cash SS, et al Dynamic balance of excitation and inhibition in human and monkey neocortex. Scientific reports. 2016;6 10.1038/srep23176 PMC4793223 26980663

[11] Monier C, Fournier J, Frégnac Y. In vitro and in vivo measures of evoked excitatory and inhibitory conductance dynamics in sensory cortices. Journal of neuroscience methods. 2008;169(2):323–365. 10.1016/j.jneumeth.2007.11.008 18215425

[12] Borg-Graham L, Monier C, Fregnac Y. Voltage-clamp measurement of visually-evoked conductances with whole-cell patch recordings in primary visual cortex. Journal of Physiology-Paris. 1996;90(3):185–188. 10.1016/S0928-4257(97)81421-0 9116665

[13] Borg-Graham LJ, Monier C, Fregnac Y. Visual input evokes transient and strong shunting inhibition in visual cortical neurons. Nature. 1998;393(6683):369–373. 10.1038/30735 9620800

[14] Atallah BV, Bruns W, Carandini M, Scanziani M. Parvalbumin-expressing interneurons linearly transform cortical responses to visual stimuli. Neuron. 2012;73(1):159–170. 10.1016/j.neuron.2011.12.013 22243754 PMC3743079

[15] Zhang LI, Tan AY, Schreiner CE, Merzenich MM. Topography and synaptic shaping of direction selectivity in primary auditory cortex. Nature. 2003;424(6945):201–205. 10.1038/nature01796 12853959

[16] Wehr M, Zador AM. Synaptic mechanisms of forward suppression in rat auditory cortex. Neuron. 2005;47(3):437–445. 10.1016/j.neuron.2005.06.009 16055066

[17] Ye Cq, Poo Mm, Dan Y, Zhang Xh. Synaptic mechanisms of direction selectivity in primary auditory cortex. The Journal of Neuroscience. 2010;30(5):1861–1868. 10.1523/JNEUROSCI.3088-09.2010 20130195 PMC2833018

[18] Shu Y, Hasenstaub A, McCormick DA. Turning on and off recurrent balanced cortical activity. Nature. 2003;423(6937):288–293. 10.1038/nature01616 12748642

[19] Haider B, Duque A, Hasenstaub AR, McCormick DA. Neocortical network activity in vivo is generated through a dynamic balance of excitation and inhibition. The Journal of neuroscience. 2006;26(17):4535–4545. 10.1523/JNEUROSCI.5297-05.2006 16641233 PMC6674060

[20] Koch C. Biophysics of computation: information processing in single neurons. Oxford university press; 2004.

[21] Carnevale NT, Johnston D. Electrophysiological characterization of remote chemical synapses. Journal of Neurophysiology. 1982;47(4):606–621. 10.1152/jn.1982.47.4.606 7069456

[22] Brown TH, Johnston D. Voltage-clamp analysis of mossy fiber synaptic input to hippocampal neurons. Journal of Neurophysiology. 1983;50(2):487–507. 10.1152/jn.1983.50.2.487 6136553

[23] Rall W, Segev I. Space-clamp problems when voltage clamping branched neurons with intracellular microelectrodes In: Voltage and patch clamping with microelectrodes. Springer; 1985 p. 191–215.

[24] Clements J, Redman S. Cable properties of cat spinal motoneurones measured by combining voltage clamp, current clamp and intracellular staining. The Journal of Physiology. 1989;409(1):63–87. 10.1113/jphysiol.1989.sp017485 2585300 PMC1190432

[25] Spruston N, Jaffe DB, Williams SH, Johnston D. Voltage-and space-clamp errors associated with the measurement of electrotonically remote synaptic events. Journal of Neurophysiology. 1993;70(2):781–802. 10.1152/jn.1993.70.2.781 8410172

[26] Williams SR, Mitchell SJ. Direct measurement of somatic voltage clamp errors in central neurons. Nature neuroscience. 2008;11(7):790–798. 10.1038/nn.2137 18552844

[27] Poleg-Polsky A, Diamond JS. Imperfect space clamp permits electrotonic interactions between inhibitory and excitatory synaptic conductances, distorting voltage clamp recordings. PLoS One. 2011;6(4):e19463 10.1371/journal.pone.0019463 21559357 PMC3085473

[28] Hao J, Wang Xd, Dan Y, Poo Mm, Zhang Xh. An arithmetic rule for spatial summation of excitatory and inhibitory inputs in pyramidal neurons. Proceedings of the National Academy of Sciences. 2009;106(51):21906–21911. 10.1073/pnas.0912022106 PMC2799885 19955407

[29] Davie JT, Kole MH, Letzkus JJ, Rancz EA, Spruston N, Stuart GJ, et al Dendritic patch-clamp recording. Nature protocols. 2006;1(3):1235–1247. 10.1038/nprot.2006.164 17406407 PMC7616975

[30] Barry PH. JPCalc, a software package for calculating liquid junction potential corrections in patch-clamp, intracellular, epithelial and bilayer measurements and for correcting junction potential measurements. Journal of neuroscience methods. 1994;51(1):107–116. 10.1016/0165-0270(94)90031-0 8189746

[31] Li S, Liu N, Zhang Xh, Zhou D, Cai D. Bilinearity in spatiotemporal integration of synaptic inputs. PLoS computational biology. 2014;10(12):e1004014 10.1371/journal.pcbi.1004014 25521832 PMC4270458

[32] Li S, Zhou D, Cai D. Analysis of the dendritic integration of excitatory and inhibitory inputs using cable models. Communications in Mathematical Sciences. 2015;13(2):565–575. 10.4310/CMS.2015.v13.n2.a16

[33] Cannon R, Turner D, Pyapali G, Wheal H. An on-line archive of reconstructed hippocampal neurons. Journal of neuroscience methods. 1998;84(1):49–54. 10.1016/S0165-0270(98)00091-0 9821633

[34] Destexhe A, Mainen ZF, Sejnowski TJ. An efficient method for computing synaptic conductances based on a kinetic model of receptor binding. Neural computation. 1994;6(1):14–18. 10.1162/neco.1994.6.1.14

[35] Destexhe A, Mainen ZF, Sejnowski TJ. Synthesis of models for excitable membranes, synaptic transmission and neuromodulation using a common kinetic formalism. Journal of computational neuroscience. 1994;1(3):195–230. 10.1007/BF00961734 8792231

[36] Poirazi P, Brannon T, Mel BW. Arithmetic of subthreshold synaptic summation in a model CA1 pyramidal cell. Neuron. 2003;37(6):977–987. 10.1016/S0896-6273(03)00148-X 12670426

[37] Poirazi P, Brannon T, Mel BW. Pyramidal neuron as two-layer neural network. Neuron. 2003;37(6):989–999. 10.1016/S0896-6273(03)00149-1 12670427

[38] Stuart G, Spruston N. Determinants of voltage attenuation in neocortical pyramidal neuron dendrites. The Journal of neuroscience. 1998;18(10):3501–3510. 10.1523/JNEUROSCI.18-10-03501.1998 9570781 PMC6793161

[39] Magee JC, Johnston D. Characterization of single voltage-gated Na+ and Ca2+ channels in apical dendrites of rat CA1 pyramidal neurons. The Journal of Physiology. 1995;487:67–90. 10.1113/jphysiol.1995.sp020862 7473260 PMC1156600

[40] Hoffman DA, Magee JC, Colbert CM, Johnston D. K+ channel regulation of signal propagation in dendrites of hippocampal pyramidal neurons. Nature. 1997;387(6636):869–875. 10.1038/43119 9202119

[41] Migliore M, Hoffman D, Magee J, Johnston D. Role of an A-type K+ conductance in the back-propagation of action potentials in the dendrites of hippocampal pyramidal neurons. Journal of computational neuroscience. 1999;7(1):5–15. 10.1023/A:1008906225285 10481998

[42] Magee JC. Dendritic hyperpolarization-activated currents modify the integrative properties of hippocampal CA1 pyramidal neurons. The Journal of neuroscience. 1998;18(19):7613–7624. 10.1523/JNEUROSCI.18-19-07613.1998 9742133 PMC6793032

[43] Magee JC, Cook EP. Somatic EPSP amplitude is independent of synapse location in hippocampal pyramidal neurons. Nature neuroscience. 2000;3(9):895–903. 10.1038/78800 10966620

[44] Andrásfalvy BK, Magee JC. Distance-dependent increase in AMPA receptor number in the dendrites of adult hippocampal CA1 pyramidal neurons. The Journal of Neuroscience. 2001;21(23):9151–9159. 10.1523/JNEUROSCI.21-23-09151.2001 11717348 PMC6763889

[45] Smith MA, Ellis-Davies GC, Magee JC. Mechanism of the distance-dependent scaling of Schaffer collateral synapses in rat CA1 pyramidal neurons. The Journal of physiology. 2003;548(1):245–258. 12598591 10.1113/jphysiol.2002.036376 PMC2342790

[46] Nicholson DA, Trana R, Katz Y, Kath WL, Spruston N, Geinisman Y. Distance-dependent differences in synapse number and AMPA receptor expression in hippocampal CA1 pyramidal neurons. Neuron. 2006;50(3):431–442. 10.1016/j.neuron.2006.03.022 16675397

[47] Carnevale NT, Hines ML. The NEURON book. Cambridge: Cambridge Univ. Press; 2006.

[48] Carandini M, Mechler F, Leonard CS, Movshon JA. Spike train encoding by regular-spiking cells of the visual cortex. Journal of Neurophysiology. 1996;76(5):3425–3441. 10.1152/jn.1996.76.5.3425 8930283

[49] Badel L, Lefort S, Brette R, Petersen CC, Gerstner W, Richardson MJ. Dynamic IV curves are reliable predictors of naturalistic pyramidal-neuron voltage traces. Journal of Neurophysiology. 2008;99(2):656–666. 10.1152/jn.01107.2007 18057107

[50] Cash S, Yuste R. Input summation by cultured pyramidal neurons is linear and position-independent. The Journal of neuroscience. 1998;18(1):10–15. 10.1523/JNEUROSCI.18-01-00010.1998 9412481 PMC6793421

[51] Cash S, Yuste R. Linear summation of excitatory inputs by CA1 pyramidal neurons. Neuron. 1999;22(2):383–394. 10.1016/S0896-6273(00)81098-3 10069343

[52] Priebe NJ, Ferster D. Direction selectivity of excitation and inhibition in simple cells of the cat primary visual cortex. Neuron. 2005;45(1):133–145. 10.1016/j.neuron.2004.12.024 15629708

[53] Wilent WB, Contreras D. Synaptic responses to whisker deflections in rat barrel cortex as a function of cortical layer and stimulus intensity. The Journal of neuroscience. 2004;24(16):3985–3998. 10.1523/JNEUROSCI.5782-03.2004 15102914 PMC6729426

[54] Sabatini B, Regehr W. Timing of synaptic transmission. Annual Review of Physiology. 1999;61(1):521–542. 10.1146/annurev.physiol.61.1.521 10099700

[55] Spruston N, Johnston D. Out of control in the dendrites. Nature neuroscience. 2008;11(7):733 10.1038/nn0708-733 18575467

[56] Pearce RA. Physiological evidence for two distinct GABAA responses in rat hippocampus. Neuron. 1993;10(2):189–200. 10.1016/0896-6273(93)90310-N 8382497

[57] Häusser M, Roth A. Estimating the time course of the excitatory synaptic conductance in neocortical pyramidal cells using a novel voltage jump method. Journal of Neuroscience. 1997;17(20):7606–7625. 10.1523/JNEUROSCI.17-20-07606.1997 9315883 PMC6793890

[58] Magee JC. Dendritic integration of excitatory synaptic input. Nature Reviews Neuroscience. 2000;1(3):181–190. 10.1038/35044552 11257906

[59] Kim SJ, Linden DJ. Ubiquitous plasticity and memory storage. Neuron. 2007;56(4):582–592. 10.1016/j.neuron.2007.10.030 18031678

[60] Beaulieu-Laroche L, Harnett MT. Dendritic Spines Prevent Synaptic Voltage Clamp. Neuron. 2018;97(1):75–82. 10.1016/j.neuron.2017.11.016 29249288

[61] Lankarany M, Heiss JE, Lampl I, Toyoizumi T. Simultaneous bayesian estimation of excitatory and inhibitory synaptic conductances by exploiting multiple recorded trials. Frontiers in computational neuroscience. 2016;10:110 10.3389/fncom.2016.00110 27867353 PMC5095134

[62] Chizhov AV, Amakhin DV. Method of experimental synaptic conductance estimation: Limitations of the basic approach and extension to voltage-dependent conductances. Neurocomputing. 2018;275:2414–2425. 10.1016/j.neucom.2017.11.017

[63] Pospischil M, Piwkowska Z, Bal T, Destexhe A. Extracting synaptic conductances from single membrane potential traces. Neuroscience. 2009;158(2):545–552. 10.1016/j.neuroscience.2008.10.033 19027831

[64] Chizhov A, Malinina E, Druzin M, Graham LJ, Johansson S. Firing clamp: a novel method for single-trial estimation of excitatory and inhibitory synaptic neuronal conductances. Frontiers in cellular neuroscience. 2014;8:86 10.3389/fncel.2014.00086 24734000 PMC3973923

[65] Vich C, Guillamon A. Dissecting estimation of conductances in subthreshold regimes. Journal of computational neuroscience. 2015;39(3):271–287. 10.1007/s10827-015-0576-2 26432075

[66] Yaşar TB, Wright NC, Wessel R. Inferring presynaptic population spiking from single-trial membrane potential recordings. Journal of neuroscience methods. 2016;259:13–21. 10.1016/j.jneumeth.2015.11.019 26658223

[67] Vich C, Berg RW, Guillamon A, Ditlevsen S. Estimation of synaptic conductances in presence of nonlinear effects caused by subthreshold ionic currents. Frontiers in computational neuroscience. 2017;11:69 10.3389/fncom.2017.00069 28790909 PMC5524927

[68] Branco T, Häusser M. The single dendritic branch as a fundamental functional unit in the nervous system. Current opinion in neurobiology. 2010;20(4):494–502. 10.1016/j.conb.2010.07.009 20800473

[69] Doron M, Chindemi G, Muller E, Markram H, Segev I. Timed synaptic inhibition shapes NMDA spikes, influencing local dendritic processing and global I/O properties of cortical neurons. Cell reports. 2017;21(6):1550–1561. 10.1016/j.celrep.2017.10.035 29117560

[70] Zhou D, Li S, Zhang Xh, Cai D. Phenomenological incorporation of nonlinear dendritic integration using integrate-and-fire neuronal frameworks. PloS one. 2013;8(1):e53508 10.1371/journal.pone.0053508 23308241 PMC3538611

[71] Atherton LA, Burnell ES, Mellor JR. Assessment of methods for the intracellular blockade of GABAA receptors. PloS one. 2016;11(8):e0160900 10.1371/journal.pone.0160900 27501143 PMC4976935

